# Genome-Wide Identification and Characterization of the *SWEET* Gene Family in *Phoebe bournei* with an Emphasis on Hormonal Responses and Plant Physiological Changes

**DOI:** 10.3390/plants15121914

**Published:** 2026-06-20

**Authors:** Xuan Wang, Cheyuan Wang, Duo Yu, Wenjing Lin, Jiaying Qian, Xinghao Tang, Kehui Zheng

**Affiliations:** 1 College of Forestry, Fujian Agriculture and Forestry University, Fuzhou 350002, China; 19359333985@163.com (X.W.); ashwcy20060601@163.com (C.W.); 15080281453@163.com (W.L.); jiayingq0104@163.com (J.Q.); txh060404009@163.com (X.T.); 2 College of Agriculture, Fujian Agriculture and Forestry University, Fuzhou 350002, China; 18661567099@163.com; 3 Fujian Academy of Forestry Sciences, Fuzhou 350012, China; 4 College of Computer and Information Sciences, Fujian Agriculture and Forestry University, Fuzhou 350002, China

**Keywords:** *P. bournei*, *SWEET* gene family, genome-wide identification, evolutionary conservation, hormone response, plant physiological responses

## Abstract

The Sugars Will Eventually be Exported Transporters (SWEET) family plays a crucial role in the carbohydrate distribution, phloem loading, and stress response of plants, yet the evolutionary characteristics and functional diversification of *SWEET* genes in the endangered timber species *Phoebe bournei* (Hemsl.) Yen C. Yang remain largely unexplored. In this study, 21 *PbSWEET* genes were identified and classified into four subfamilies (A–D). Subfamily A exhibited a unique lineage expansion, mainly driven by tandem and segmental duplications. The nonsynonymous-to-synonymous substitution ratio (Ka/Ks) values of all duplicate gene pairs were all less than 1, indicating a strong selective suppression effect; consistent with this evolutionary constraint, the majority of PbSWEET proteins harbor the conserved *Medicago truncatula* Nodulin 3/saliva (MtN3_slv) domain, with only a few exceptions lacking a complete version. Promoter and hormone response analyses revealed that under abscisic acid (ABA) stress, *PbSWEET4* exhibited an immediate burst, whereas *PbSWEET10* showed a delayed burst. Physiological data indicated that soluble sugars may be more dominant osmolytes than proline (Pro), a pattern that points to a potential carbon-centric regulatory strategy. *PbSWEET4* showed an early burst before sugar/oxidative peaks, suggesting a possible non-canonical signaling role, whereas *PbSWEET10* exhibited a late increase coinciding with sugar/malondialdehyde (MDA) peaks, suggesting potential involvement in sugar redistribution. Under methyl jasmonate (MeJA) treatment, *PbSWEET10* was rapidly induced, yet sugar accumulation occurred only at 24 h, a temporal decoupling that suggests a possible transcription–metabolism decoupling. Collectively, these correlative patterns point to a possible dual-wave transcriptional mechanism and nominate *PbSWEET10* as a candidate for stress response, though these inferences require functional validation.

## 1. Introduction

Sugars serve as the energy foundation and structural components of plant life activities, and their transmembrane transport is crucial for cellular functions, growth, development, and environmental adaptation [1,2,3,4,5]. In plants, sugar allocation relies on the precise regulation of a series of sugar transporters, which mediate long-distance transport between source and sink organs as well as intracellular compartmentalization, thereby supporting key physiological processes, including photoassimilate partitioning, seed filling, and fruit development [1,6,7]. In recent years, the Sugars Will Eventually be Exported Transporters (SWEET) family, a new type of transport sugar protein, has received considerable attention because of its unique role. They belong to the *Medicago truncatula* Nodulin 3/saliva (MtN3_slv) superfamily. This class of proteins typically contains two conserved *M*. *truncatula* Nodulin 3 (MtN3) domains, forming a membrane topology with seven transmembrane helices, and these are low-affinity, high-capacity bidirectional transport proteins that help sugars pass through the cell membrane [8]. SWEET proteins play significant roles in phloem loading, nectar secretion, nutrient supply to pollen tubes, and seed development, and have also been suggested to participate in cytokinin transport [7,8,9]. Particularly important is the pivotal role of the SWEET family in plant interactions with biotic and abiotic environments. Research has demonstrated that SWEET-mediated sugar outflow is a crucial interface in carbon allocation and plays a major part in plant–microbe interactions; for example, pathogenic microbes can use them to obtain host sugars and alter plant susceptibility or resistance [10,11,12,13]. Meanwhile, by controlling the buildup and distribution of soluble sugars, SWEET members actively participate in plant responses to environmental challenges, including drought and low temperature, to assist in the maintenance of cellular osmotic balance and energy homeostasis [14,15]. Therefore, the evolutionary characteristics and functional changes of the *SWEET* gene family can help us better understand how plants transport sugar and the molecular mechanism of sugar transport in the plant’s adaptation environment [1,7,8].

Growing evidence confirms the integral role of these transporters in key physiological processes, including plant growth and development, host–pathogen interactions, and responses to abiotic stresses [10,16,17]. For example, in the classical field of sugar transport, in *Oryza sativa* L. (rice), OsSWEET11 and OsSWEET15 have been confirmed as key regulatory factors in rice grain filling, mediating efficient sucrose unloading from maternal tissues to the endosperm via the apoplastic pathway, thereby ensuring normal seed development [18].

In addition to these well-established roles in sugar allocation, an increasing number of SWEET proteins have been found to transport non-sugar substrates, including phytohormones. In *Arabidopsis thaliana* (L.) Heynh, AtSWEET13 and AtSWEET14 have been discovered to transport gibberellic acid (GA), and they can also be used to help GA-mediated processes such as anther dehiscence and seed development [19,20]. This pioneering discovery reveals a new role of SWEET proteins as hormone transport carriers, greatly expanding the boundaries of their functional research. Furthermore, the function of *SWEET* genes in response to hormonal signals has increasingly attracted attention. Among monocotyledonous herbaceous plants, studies on maize (*Zea mays* L.) and *Lilium* ‘Sorbonne’ (Oriental hybrid lily) have revealed the effect of the precise regulation of abscisic acid (ABA) signaling on the expression of *SWEET* genes: In maize, members of the maize *SWEET* gene family are rich in ABA-responsive elements, their expression being induced by exogenous ABA, and genes such as *ZmSWEET1a* respond simultaneously to drought, saline–alkaline stress and ABA treatment, suggesting that they may participate in abiotic stress responses through ABA-dependent signaling pathways [21]; *LoSWEET14* in *L.* ‘Sorbonne’ reveals a direct regulatory pathway from hormone signaling to gene expression, where the transcription factor LoABF2 can directly bind to the ABA response element in its promoter, activating *LoSWEET14* expression and subsequently responding to various abiotic stresses by promoting soluble sugar accumulation [22]. In dicotyledonous herbaceous plants, such as *Nicotiana tabacum* L., the overexpression of *NtSWEET12i* can promote the transport of soluble sugars and significantly enhance plant tolerance to drought and saline–alkaline stress by activating the ABA signaling pathway [23]. Similarly, in dicotyledonous herbaceous vine plants, in *Passiflora edulis* Sims, *PeSWEET3* has been proven to have ABA-responsive elements in its promoter, and exogenous ABA treatment can significantly upregulate its expression and promote the accumulation of soluble sugars, directly establishing the link between ABA signaling and SWEET-mediated sugar accumulation [24]. Research on a dicotyledonous woody plant, *Camellia oleifera* Abel, is more in-depth; *CoSWEET2a* not only maintains cellular sugar homeostasis to enhance drought resistance but its expression is also significantly induced by both ABA and GA, demonstrating the fine regulation of *SWEET* function by hormonal signals at the transcriptional level [25]. Furthermore, recent studies have found that ABA-induced seed germination can coordinately regulate the biosynthesis of cuticular wax and cuticle by activating endogenous ABA and jasmonic acid (JA) signaling pathways to enhance drought tolerance. This supports the idea that hormone signaling can reshape carbon allocation under stress, raising the possibility that ABA or methyl jasmonate (MeJA) may directly regulate *SWEET* expression [26]. In summary, the functions of the SWEET family have expanded from mere sugar transport to active interplay with hormone signaling networks (especially ABA, MeJA, and GA). Some SWEETs directly transport hormones, whereas others serve as downstream effectors of hormone signaling; both roles allow SWEETs to finely modulate plant growth, development, and stress adaptation by controlling sugar distribution and accumulation.

Despite these advances in model plants and major crops, research on the *SWEET* gene family in forest trees remains remarkably limited. *Phoebe bournei* (Hemsl.) Yen C. Yang is a near-threatened timber tree species of significant ecological and commercial value in China. Its excellent wood quality and high decay resistance make it popular in forestry and landscape building [27]. However, environmental pressures, particularly frequent droughts and soil microplastic contamination caused by global climate change, have seriously threatened the natural distribution and long-term management of *P. bournei* plants [28,29]. Recent studies have shown that *P. bournei* can respond to such stresses through complex physiological and molecular processes; drought stress, for example, significantly reduces photosynthesis, activates antioxidant systems, and modifies hormone signaling pathways such as ABA [29,30,31]. These adaptive responses are frequently accompanied by the accumulation and redistribution of photoassimilates [30], such as soluble sugars, implying that sugar transporters may play an important role in these processes, and their potential role, especially in the ABA and MeJA signaling pathways, has not been explored yet. As a major subfamily of sugar transporters, SWEETs are prime candidates to explore this role. However, the distribution, evolutionary features, and functional roles under stress conditions of the *SWEET* family remain systematically unexplored in *P. bournei*.

To close this knowledge gap, this project identified and analyzed the *SWEET* gene family across the genome in *P. bournei*. Drawing on the strategies used in previous studies, we conducted a multi-dimensional analysis of the *SWEET* family, covering its evolutionary dynamics, structural features, and transcriptional responses to hormones [32,33,34,35]. These analyses, together with physiological indicator measurements, are expected to clarify the evolutionary relationships of *PbSWEET* members and reveal their potential roles in abiotic stress adaptation, including how they may contribute to key physiological processes such as osmotic regulation and oxidative stress mitigation, particularly within hormone-mediated signaling pathways [36]. The results of this study may also provide a theoretical basis for further exploration of the potential role of this family in plant–microbe interactions [37] and sugar allocation [38,39]. This study aims to provide key genetic resources for clarifying the molecular basis of sugar signaling and transport in *P. bournei*, thereby offering candidate genes and a theoretical foundation for the genetic improvement of stress resistance in forest trees.

## 2. Results

### 2.1. Identification and Physicochemical Property Analysis of PbSWEET Genes in P. bournei

*P. bournei* was found to have 21 *SWEET* genes, which were renamed *PbSWEET1* through *PbSWEET21* (Table 1). The average length of the encoded proteins is roughly 244 aa, with all lengths ranging from 68 aa to 439 aa. The molecular weights of the PbSWEET proteins show significant variation in protein size within this family, ranging from 7.87 kDa (PbSWEET4) to 50.02 kDa (PbSWEET11), with an average of roughly 27.104 kDa. With theoretical isoelectric points (pIs) ranging from 7.58 to 9.64, all 21 PbSWEET proteins are categorized as basic proteins. In terms of protein stability, it is predicted that 13 proteins (PbSWEET1, PbSWEET2, PbSWEET4, and others with an instability index < 40) will be stable, while the remaining eight are unstable (instability index > 40), with all values ranging from 30.05 to 46.45. The aliphatic index of the PbSWEET proteins has a mean value of 114.64, and its range is from 90.29 to 127.69, which suggests the proteins generally have strong heat stability. Also, all grand average of hydropathicity (GRAVY) values are positive with the exception of PbSWEET11 (−0.178), with the maximum GRAVY value being 0.987 (PbSWEET7), which means the PbSWEET proteins are hydrophobic for the most part. Meanwhile, subcellular localization was predicted. Most PbSWEET proteins (16) are located in the plasma membrane, supporting their core role in transmembrane sugar transport. Three proteins (PbSWEET3, PbSWEET4, and PbSWEET13) are found in the chloroplast, and two proteins (PbSWEET6 and PbSWEET9) are located in the vacuole.

### 2.2. Chromosome Localization and Evolutionary Analysis of PbSWEET Genes in P. bournei

**Figure 1:**
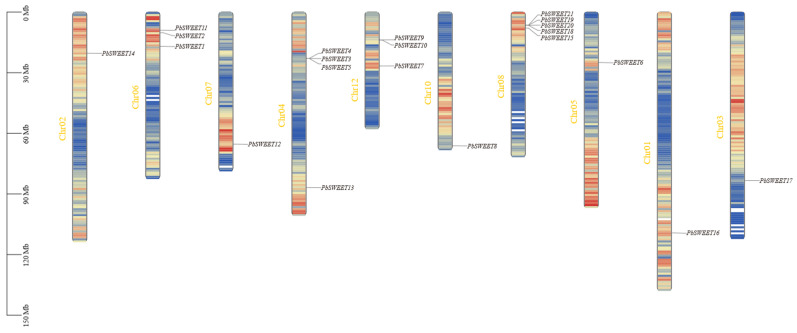
A schematic diagram showing the distribution of the *PbSWEET* genes in the chromosomes (Chr) of *P. bournei*; the scale on the left can be used to assess chromosomal length and gene position.

To ascertain the chromosomal locations of 21 *PbSWEET* genes in *P. bournei*, we conducted a location map that shows they were located unevenly across 10 chromosomes (Figure 1). We found that chromosome (Chr) 08 contains the largest number of this gene family (a total of five), and these genes are arranged closely together. Similarly, three genes on Chr04 were also densely distributed. The clustered distribution pattern of these genes conforms to the typical pattern of tandem repeats. Six out of the ten chromosomes carried only one *PbSWEET* gene each, and this single-gene distribution pattern could conform to the characteristics of whole-genome or large-scale replication events. Chr04, Chr06, and Chr12 harbored four, three, and three *PbSWEET* genes, respectively. On these chromosomes, the genes were found in both tight clusters and as single, scattered genes. This coupling distribution pattern also conforms to the characteristic of multiple evolutionary models acting simultaneously.

**Figure 2:**
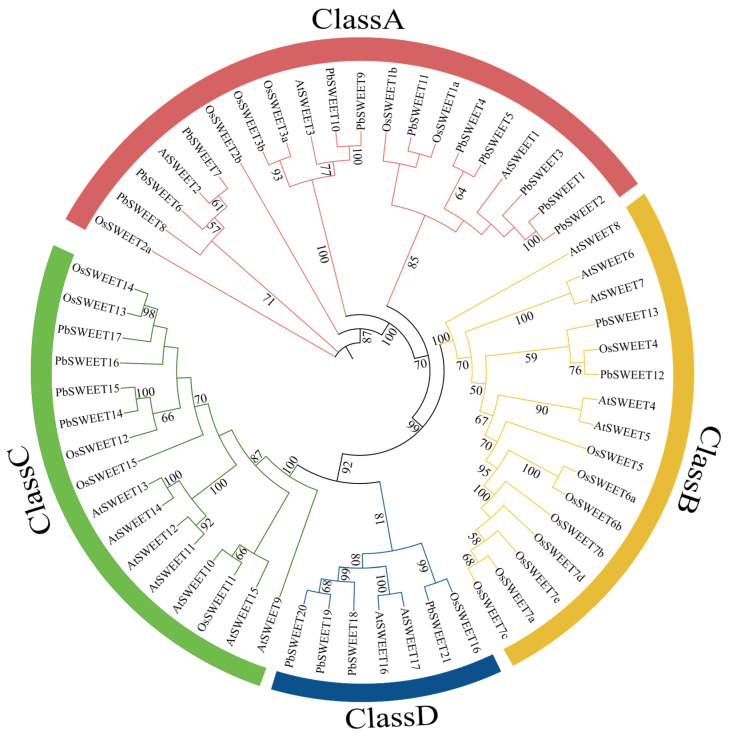
The evolutionary tree of SWEET proteins among the three plant species (21 proteins from *P. bournei*, 17 from *A. thaliana*, and 21 from *O. sativa*, respectively). The tree was constructed using the Maximum Likelihood method implemented in MEGA11 software, with 1000 bootstrap replicates. The colored arcs represent different subfamilies; among them, type A is represented by red, type B by yellow, type C by green, and type D by blue. The numbers at the branches represent bootstrap support values.

Furthermore, we constructed an evolutionary tree using 21 proteins from *P. bournei*, 17 from *A. thaliana*, and 21 from *O. sativa*, totaling 59 proteins (Figure 2). These proteins were grouped into four subfamilies, named A through D. Subfamilies A, B, and C had an approximately similar number of members, while the D subfamily is relatively small, containing only seven members of the SWEET protein. As can be seen from the figure, among the *PbSWEET* genes, subfamily A was the largest, containing 11 members, accounting for 52.38% of all *PbSWEET* genes; subfamily B was the smallest, containing only two members, *PbSWEET12* and *PbSWEET13*, accounting for 9.52%. This pattern shows the *PbSWEET* family expanded in an unbalanced way during evolution; subfamily A appears to be the core amplified group, while subfamily B seems to have followed a more conserved evolutionary path. Moreover, the figure also shows that the number of *SWEET* genes in different species varies significantly across subfamilies.

### 2.3. Gene Structure, Conserved Motifs, and Conserved Domain Analysis of PbSWEET Genes in P. bournei

**Figure 3:**
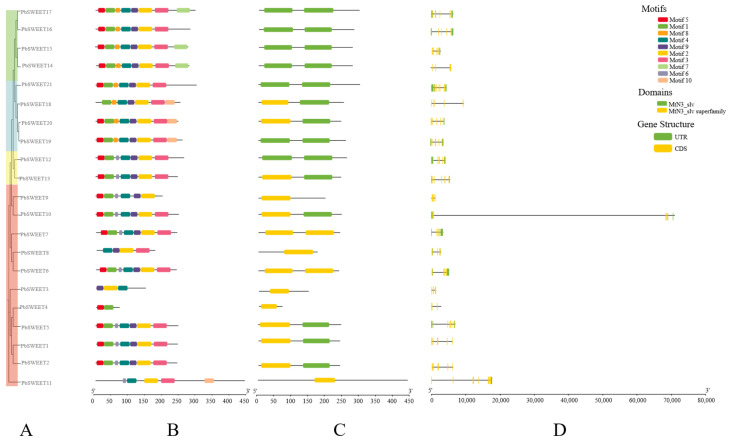
The gene structure, conserved motifs, and conserved domains of *PbSWEET* genes in *P. bournei*. (**A**) Evolutionary analysis of PbSWEET proteins was constructed using the maximum likelihood algorithm, using bootstrap with 1000 replications. (**B**) The distribution of conserved motifs on the PbSWEET proteins. (**C**) Analysis of the conserved domains in the PbSWEET proteins. (**D**) The gene structure of the *PbSWEET* gene family.

To clarify the structural diversity and evolutionary conservation of the *PbSWEET* gene family, we analyzed the exon–intron structures of 21 *PbSWEET* genes (Figure 3D). This analysis showed great variety in gene length and implied great diversity in the arrangement configuration of the exons and introns, simultaneously reflecting the evolutionary flexibility of this gene family. Nearly half of the genes (*PbSWEET1*, *PbSWEET2*, *PbSWEET3*, *PbSWEET4* and *PbSWEET9*) in the A subfamily lacked untranslated regions (UTRs), perhaps suggesting a simpler regulatory network in this subfamily’s work. Notably, *PbSWEET4* lacks UTRs but encodes a truncated protein (68 aa) that is unlikely to function as a canonical sugar transporter; its biological significance remains to be determined. The B subfamily contained only two genes (*PbSWEET12* and *PbSWEET13*), both of which showed a classic structure with dispersed exons, which suggests they evolved with high conservation and their functions are also likely very similar. Additionally, the presence of UTRs in these genes could indicate that they can reach precise regulation in work. The C and D subfamilies both showed notable differences in their internal structures, demonstrating that they both have a pattern of separating evolution in their internal structures, and such divergence supports the idea that they have developed diverse functions.

Furthermore, we examined 10 conserved motifs in the 21 PbSWEET proteins (Figure 3B). Most members shared similar sets and orders of these motifs, strongly indicating there could be evolutionary conservation and consistent function within the family. However, Motif 7 was found only in the C subfamily. This exclusive presence could suggest a specialized role for this subfamily. Analysis of protein domains showed clear patterns (Figure 3C). The A subfamily primarily contained domains from the MtN3_slv superfamily, while the B, C, and D subfamilies mainly consist of MtN3_slv domains. Detailed domain coordinates and E-values for each protein are provided (Table S1).

It is worth noting that among the 21 PbSWEET proteins, PbSWEET4 is particularly short, containing only 68 amino acid residues (Table 1). This length is much shorter than the typical length of the SWEET protein. To assess whether this short peptide can fold into a functional transporter protein, we performed homology modeling (Figure S1). The obtained model shows a local structural similarity (with an identity of 48.33%), but the coverage is extremely low (approximately 20–30%), and there are discontinuous alignments, making it impossible to construct a complete MtN3_slv domain or a typical seven-span membrane topology structure. These observations indicate that although PbSWEET4 belongs to the MtN3_slv superfamily based on sequence analysis, it is unlikely to function as an independent sugar transporter.

### 2.4. Cis-Acting Elements Analysis of PbSWEET Genes in P. bournei

**Figure 4:**
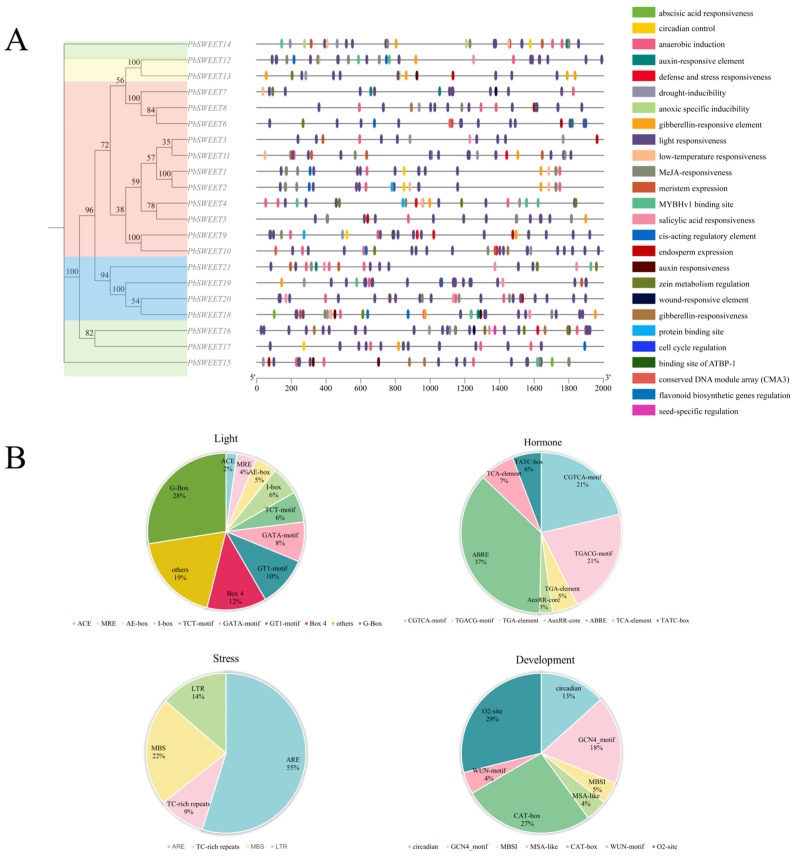
*Cis*-acting elements analysis in *PbSWEET* promoters. (**A**) Classification and distribution of predicted *cis*-regulatory elements in the promoter regions of the *PbSWEET* genes. (**B**) The pie chart shows the relative abundance of different *cis*-regulatory elements, and these elements are classified into four major response types: plant hormone response elements, stress response elements, light response elements, and developmental-related elements.

In order to explore the potential regulatory mechanisms underlying the expression and function of the *PbSWEET* genes, we predicted and analyzed the region that is located 2000 bp upstream of each *PbSWEET* gene, identifying a total of 26 types of *cis*-acting elements (Figure 4). These elements are related to mechanisms such as light response, hormone signal transduction, stress adaptation, and developmental regulation. In the category of light response, the abundance of G-Box (CACGTG core motif) elements is the highest, accounting for 0.28; in terms of hormone regulation, the ABA-responsive element (ABRE) is the most abundant, with an abundance of 0.37, and the study also found two MeJA response motifs (CGTCA and TGACG), with a total abundance of 0.42; among the stress-related elements, the anaerobic induction element (ARE) sequence involved in anaerobic reactions is the most significant, with an abundance of 0.55; in the developmental regulatory elements, the abundance of the CAT-box motif, which is related to the activity of the meristem, is the highest, at 0.27.

Among different element categories, photoreceptor elements are the most numerous, followed by ABA elements and MeJA elements. The analysis could suggest the gene family is primarily involved in light response and also plays a key role in hormone signaling. Notably, some elements (seed-specific and endosperm expression elements) had very low abundance.

This predictive distribution could suggest that hormone signal transduction and stress response are the most critical mechanisms driving the expression regulation of this gene family.

### 2.5. Intraspecific Collinearity Analysis and Pairwise Evolutionary Selection Pressure Analysis of PbSWEET Genes in P. bournei

**Figure 5:**
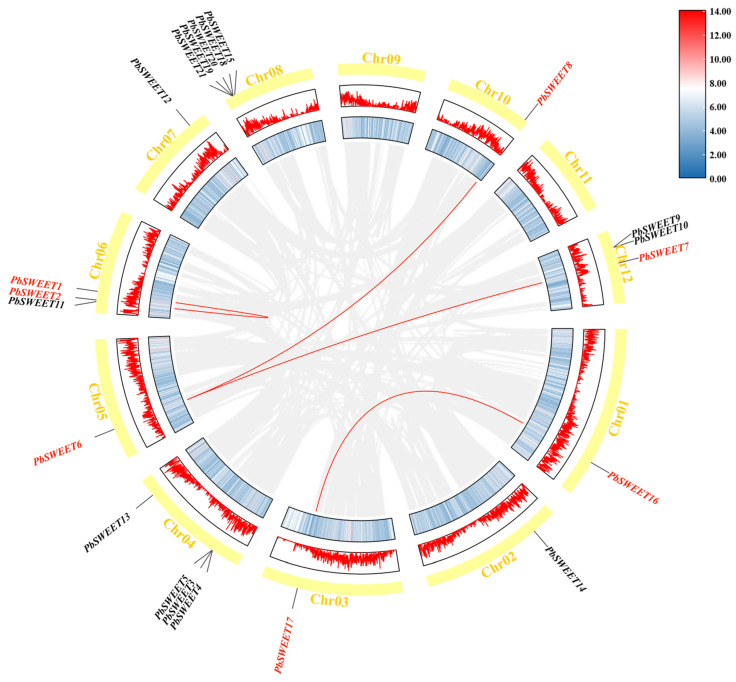
Analysis of inter-chromosomal and intra-chromosomal segment duplications of the *SWEET* genes in *P. bournei*. The gray lines represent all synthetic blocks; the red lines specifically highlight the duplicate pairs among the 21 *PbSWEET* genes.

To investigate the duplication events contributing to maintenance and evolutionary optimization of the *PbSWEET* gene family, we analyzed duplication events in the *PbSWEET* genes of *P. bournei* (Figure 5). We specifically identified four pairs of segmental duplication events, occurring between Chr01 and Chr03, Chr05 and Chr10, Chr05 and Chr12, and within Chr06. The finding could indicate that segmental duplication serves as a significant driving force for the emergence of new genes and the functional evolution of this gene family.

To further investigate the evolutionary constraints imposed on these segment-copying genes, we conducted a pairwise nonsynonymous-to-synonymous substitution ratio (Ka/Ks) analysis on them (Table 2). All examined gene pairs had a Ka/Ks ratio that was less than 1, which conforms to the evolutionary characteristics of purifying selection. For example, the ratio for *PbSWEET6/PbSWEET8* was 0.261, the ratio for *PbSWEET6/PbSWEET7* was 0.259, and the ratio for *PbSWEET17/PbSWEET16* was 0.307. Notably, although the nonsynonymous substitution rate (Ka) and synonymous substitution rate (Ks) values of *PbSWEET1* and *PbSWEET2* are both zero, which might imply genome annotation redundancy, the chromosome localization results clearly show that these two genes are located at different positions (Figure 1), thereby confirming that they are two independent gene duplication copies rather than a redundant annotation. A likely explanation is that these two genes originated from a very recent gene duplication event and have not yet accumulated any nucleotide substitutions.

### 2.6. Interspecific Collinearity Analysis of SWEET Genes in P. bournei, A. thaliana, and O. sativa

**Figure 6:**
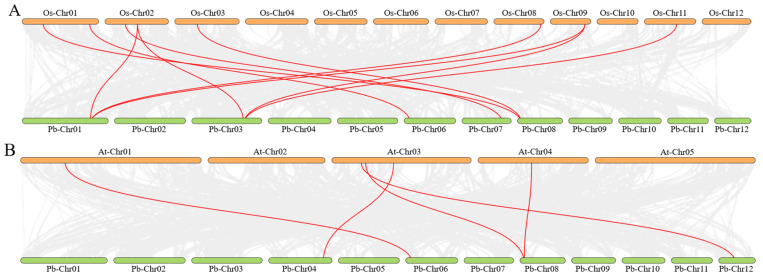
(**A**) Synteny analysis between the genomes of *P. bournei* and *O. sativa*. (**B**) Synteny analysis between the genomes of *P. bournei* and *A. thaliana*. Gray lines indicate collinear blocks within *P. bournei* and other plant genomes; the red line indicates collinear gene pairs.

To investigate the evolutionary relationships of *SWEET* genes among different species, we compared *SWEET* genes from *P. bournei* with those from *A. thaliana* (typical dicot) and *O. sativa* (representative monocot) (Figure 6). The results showed that seven *PbSWEET* genes showed considerable collinearity with genes in *O. sativa,* while four *PbSWEET* genes showed less collinearity with genes in *A. thaliana*. We also revealed specific numbers of collinear gene pairs, namely, 10 collinear gene pairs between *P. bournei* and *O. sativa* and five collinear gene pairs between *P. bournei* and *A. thaliana*.

The above results could imply that the *SWEET* genes in *P. bournei* and *O. sativa* share higher structural and historical similarity, but only within a certain level of confidence. The above analysis is reasonable because the level of collinearity is not directly equivalent to the phylogenetic relationship or the genetic evolutionary distance; the phenomena we have observed may be influenced by a variety of factors, including genomic rearrangement, gene loss, and gene duplication events [40].

### 2.7. Tissue-Specific Expression of PbSWEET Genes in P. bournei

**Figure 7:**
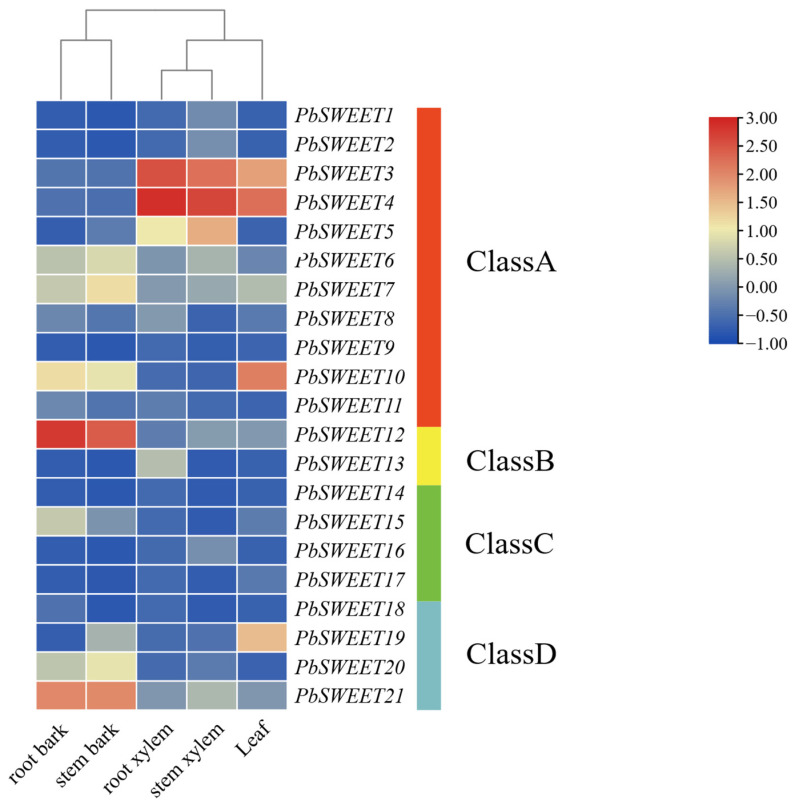
The expression patterns of 21 *PbSWEET* genes in different tissues of *P. bournei*. Expression values are shown as log_2_(FPKM + 1). The color scale bar at the top right represents log_2_(FPKM + 1) values ranging from −1 to 3. The red part on the left indicates high expression levels, while the blue part on the left represents low expression levels.

To reveal the expression specificity of the *PbSWEET* gene family in different tissues of *P. bournei* and to explore its potential division of labor in the process of sugar transport and distribution, we detected the expression levels of the members of this family in five major tissues: root bark, stem bark, root xylem, stem xylem, and leaves (Figure 7). Most genes were underexpressed or not expressed in all five tissues. The A subfamily had multiple genes that showed highly tissue-specific expression patterns; for example, *PbSWEET4* and *PbSWEET3* had very high expression in plant xylem; meanwhile, some genes in the A subfamily showed moderate expression; *PbSWEET7* and *PbSWEET6* were expressed across multiple tissues. In subfamily B, *PbSWEET12* was mainly expressed in the root bark, while *PbSWEET13* was underexpressed or not expressed in any tissue. The C subfamily showed weak expression or no expression in all tested tissues, which implies strict regulatory constraints or condition-specific induction. In contrast, the D subfamily had a more diverse expression profile.

### 2.8. GO Enrichment Analysis and Prediction of PPI Network of PbSWEET Genes and Their Encoded Proteins in P. bournei

**Figure 8:**
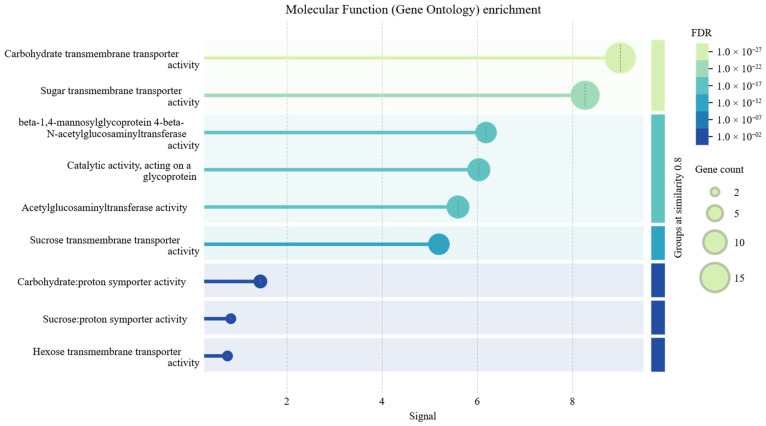
Gene Ontology (GO) enrichment analysis of molecular functions for *PbSWEET* genes. The vertical axis represents the enriched GO molecular function terms (arranged from top to bottom according to FDR from low to high), while the horizontal axis represents the enrichment signal values (higher values indicate higher enrichment levels of the function terms).

To further clarify the biological functions and molecular roles of the *PbSWEET* gene family, we conducted a Gene Ontology (GO) enrichment analysis (Figure 8). The results showed this family is mainly linked to carbohydrate transport across membranes, of which the strongest signals were for carbohydrate transmembrane transporter activity, whose term had a false discovery rate (FDR) value of 1.0 × 10^−27^. Another strong signal was for sugar transmembrane transporter activity with an FDR of 1.0 × 10^−22^. Meanwhile, some genes were also enriched for glycosyltransferase activity, namely beta-1,4-mannosylglycoprotein 4-beta-N-acetylglucosaminyltransferase activity with an FDR of 1.0 × 10^−17^, which could imply a potential role in modifying glycoproteins. Additionally, we also identified functions related to proton-coupled sugar transport, and these include sucrose–proton cotransport activity and hexose transmembrane transporter activity, which match the already known functions of the *SWEET* gene family [8,41,42].

**Figure 9:**
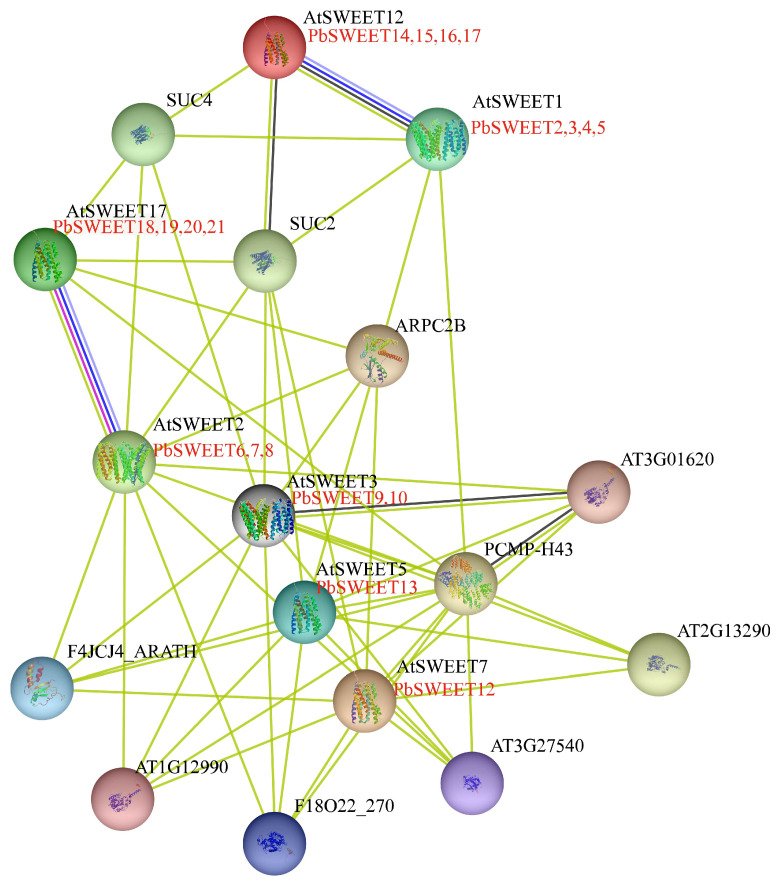
The interaction network of PbSWEET proteins was constructed by using the STRING database and the known homologous proteins of *A. thaliana*. Each node represents all the protein variants of the same gene, and these variations may arise from a series of different RNA splicing or protein modifications. The lines connecting two nodes are called edges. Light green lines represent text mining; dark blue lines denote gene co-occurrence; pink lines represent experimentally determined interactions; light purple lines indicate protein homology; and black lines represent co-expression.

After clarifying the functional characteristics of the *PbSWEET* gene family through GO enrichment analysis, we further explored the regulatory interactions among these proteins using protein–protein interaction (PPI) network analysis (Figure 9).

Network analysis showed that SWEET family members exhibited high connectivity and interactions with a variety of chaperone-related proteins, including glycosyltransferases like AT2G13290, sucrose transporters like SUC2 and SUC4, cytoskeleton-associated components like ARPC2B, and pentatricopeptide repeat (PPR) family members like PCMP-H43. It suggests that PbSWEET may cooperate with sucrose transporters in interactions or use the cytoskeleton to play a major role in sugar transfer. Meanwhile, combined with the analysis of interaction breadth and functional span, we speculate that AtSWEET3 (PbSWEET9/10) is the hub protein of the interaction network, but this speculation still needs to be verified by quantitative indices and functional experiments.

Sucrose transporter (SUC) family transporters directly interact only with SWEET family proteins in the network, suggesting that PbSWEET may be involved in the transmembrane transport of sucrose in synergy with the SUC family. Meanwhile, it suggests that PbSWEET may act as an intermediary node to indirectly connect the SUC sucrose transporter with cytoskeletal transport and glycosylation modification. Furthermore, ARPC2B interacts with multiple SWEET proteins as a connecting node, suggesting that SWEET may complete intracellular transport through ARPC2B with the assistance of the cytoskeleton to achieve transport function [43]. Although glycosyltransferase is located at the edge of the network, it is still connected to a variety of SWEET proteins. We hypothesize that PbSWEET may form a protein complex with glycosyltransferase, which is synergistic and participates in glycosylation modification-related processes, and then participates in the maintenance of plant homeostasis. Additionally, PCMP-H43, a PPR protein implicated in mitochondrial/chloroplasts and participating in RNA processing and energy metabolism regulation [44], appeared in this network, indicating that PbSWEET-mediated sugar transport may be functionally coupled with intracellular energy metabolism [45], but the specific mechanism between the two still needs to be further verified experimentally.

### 2.9. Hormone-Induced Expression Analysis of PbSWEET Genes in Phoebe bournei

**Figure 10:**
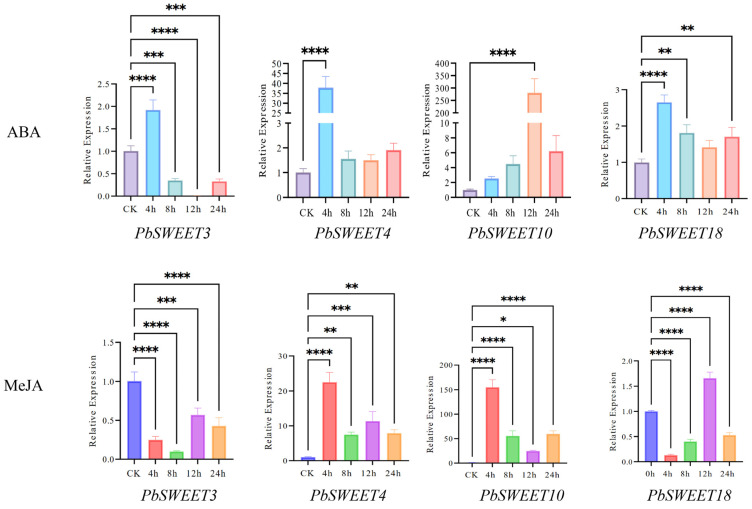
The expression patterns of *PbSWEET3, PbSWEET4*, *PbSWEET10,* and *PbSWEET18* under abscisic acid (ABA) and methyl jasmonate (MeJA) treatments. Using *PbEF1α* as the internal reference gene, qRT-PCR was employed to detect the relative expression levels of the genes, and normalization was performed with the 0 h control group set as 1. The data were presented as the mean ± standard deviation of three biological replicates (each with three technical replicates). One-way ANOVA followed by Dunnett’s post hoc test was used to determine significant differences between each treatment group and the 0 h control group at each time point (* *p* < 0.05; ** *p* < 0.01; *** *p* < 0.001; **** *p* < 0.0001; without asterisks indicating no significant difference).

To verify the expression patterns of the four candidate genes (*PbSWEET3*, *PbSWEET4*, *PbSWEET10*, and *PbSWEET18*) (screened based on *cis*-regulatory elements and tissue expression profiles) in the leaf tissues under ABA and MeJA treatments, we conducted qRT-PCR analysis (Figure 10).

Overall, this result supports the existence of distinct member-specific and time-dependent response patterns in the expression of the *PbSWEET* genes under ABA and MeJA treatment conditions. *PbSWEET4* exhibited the most rapid positive response, peaking at 4 h under both ABA and MeJA treatments. At this time point, its expression was induced approximately 38-fold by ABA and 23-fold by MeJA, after which expression levels decreased. *PbSWEET10* exhibited distinct hormone-specific delayed response characteristics under ABA treatment: It rapidly increased by approximately 155 times at 4 h after MeJA treatment, while under ABA treatment, its expression peak was delayed until 12 h, and the expression level skyrocketed to approximately 281 times that of the control. In contrast, *PbSWEET18* showed only a weak early response to ABA treatment (upregulated by approximately 2.65 times at 4 h), while its expression level was generally lower than that of the control (except for 12 h) in the presence of MeJA treatment. The expression of *PbSWEET3* was generally inhibited by the two types of hormones; at all the tested time points, the expression level was lower than the control level (except for a slight and weak increase in ABA at 4 h). Among them, *PbSWEET10* exhibited extremely significant delayed burst-like induction expression during the long-term ABA stress exposure, together with its high expression in leaves (Figure 7), thus becoming the primary candidate gene for further exploration of ABA-mediated sugar transport mechanisms under abiotic stress in *P. bournei*.

Furthermore, based on the results of the one-way ANOVA, both the ABA treatment and the MeJA treatment significantly affected the expression levels of the *PbSWEETs*. It is worth noting that both *PbSWEET4* and *PbSWEET10* exhibited sustained upregulation under both ABA and MeJA treatments, with a more pronounced and longer-lasting effect under MeJA treatment, suggesting that these two *PbSWEET* genes respond more persistently to MeJA signaling than to ABA signaling.

### 2.10. Time-Dependent Physiological Responses of P. bournei Leaves to ABA and MeJA Treatments

**Figure 11:**
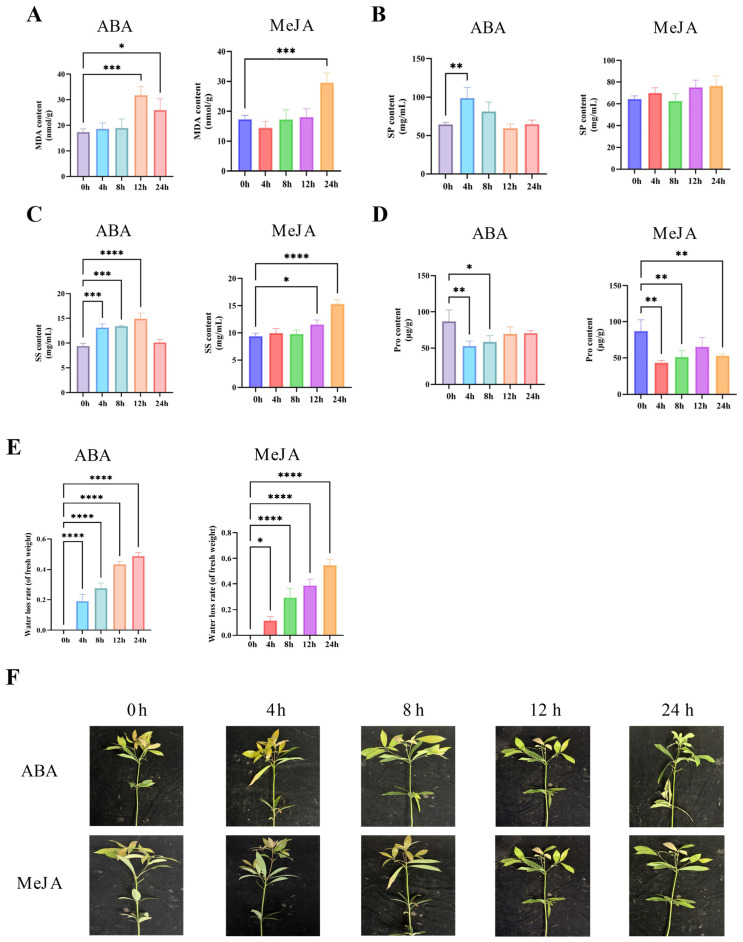
Changes in five physiological and biochemical indices and phenotypic characteristics of *P. bournei* leaves under ABA and MeJA treatments. (**A**) Changes in malondialdehyde (MDA) content. (**B**) Changes in soluble protein (SP) content. (**C**) Changes in soluble sugar (SS) content. (**D**) Changes in proline (Pro) content. (**E**) Changes in leaf water loss rate. (**F**) Changes in phenotypic characteristics. The data were presented as the mean ± standard deviation of three biological replicates (each with three technical replicates). One-way ANOVA followed by Dunnett’s post hoc test was used to determine significant differences between each treatment group and the 0 h control group at each time point (* *p* < 0.05; ** *p* < 0.01; *** *p* < 0.001; **** *p* < 0.0001; without asterisks indicating no significant difference).

To investigate the dynamic physiological regulation of *P. bournei* leaves under the induction of ABA and MeJA, we measured the leaf water loss rate, proline (Pro) (a compatible osmolyte involved in osmotic adjustment) content, malondialdehyde (MDA) (a biomarker of membrane lipid peroxidation) content, soluble sugar content and soluble protein content at 0, 4, 8, 12 and 24 h after treatment with ABA (2 mmol/L) or MeJA (2 mmol/L), with three biological replicates at each time point (each with three technical replicates) (Figure 11).

Under ABA treatment, the overall leaf water loss rate showed a continuous upward trend, reaching approximately 20% within just 4 h, but the rate of increase slowed after 12 h (Figure 11E). The contents of soluble sugars, soluble proteins, and MDA all increased first and then decreased, but the timing of the peaks differed: Soluble sugars peaked at 12 h and then dropped rapidly, suggesting dynamically regulated accumulation, possibly as a means of preventing excessive carbon consumption (Figure 11C); MDA also rapidly recovered after reaching its peak at 12 h, indicating that although ABA treatment temporarily induced membrane lipid peroxidation, it did not cause sustained oxidative damage (Figure 11A); soluble protein peaked at 4 h (about 1.50 times that of the control), a pattern consistent with the possibility that early ABA treatment may have rapidly induced the synthesis of stress-related proteins, while the subsequent continued decline suggested these proteins may be rapidly turned over or degraded (Figure 11B). Notably, the trend of Pro content was reversed, reaching its lowest point at 4 h (about 70% of the control) and then slowly recovering (Figure 11D), contrasting with the significant increase in soluble sugars, suggesting that under ABA stress, soluble sugars may serve as the main osmolyte rather than Pro.

Under MeJA treatment, the increase in leaf water loss rate was lower than the ABA level at 4 h, but then continued to rise rapidly, reaching approximately 55% in 24 h (Figure 11E), surpassing the ABA group. The responses of soluble sugars and MDA were significantly delayed. Soluble sugar content peaked at 24 h (about 1.50 times that of the control), while the change was minimal between 4 and 12 h (Figure 11C), indicating that MeJA-induced sugar accumulation initiated more slowly than ABA-induced accumulation. MDA content remained low until 24 h and only increased significantly at 24 h (about 1.50 times that of the control) (Figure 11A), indicating that early MeJA treatment did not cause significant membrane lipid peroxidation. Similar to ABA treatment, Pro slowly rebounded after reaching its lowest point at 4 h (about 50% of the control), but remained below the control (Figure 11D), further supporting the hypothesis that soluble sugars may serve as the main osmolyte in *P. bournei* in response to hormone stress. In contrast, soluble protein content fluctuated only slightly throughout the treatment period, with no early dramatic changes as observed under ABA treatment (Figure 11B).

These results suggest that ABA and MeJA activate distinct temporal programs of physiological adjustment in *P. bournei* leaves.

## 3. Discussion

The SWEET sugar transporter family has been shown to be involved in basic physiological processes, such as the distribution of plant photosynthetic products and reproductive development in herbaceous plants [1,6,7,46], and it also plays an important role in biotic stress [10,11,12,13] and abiotic stress [18,19]. However, there are differences in the life cycle, source and reservoir dynamics, and sugar transport distance between herbaceous and woody plants, so the functional understanding of the SWEET family in herbaceous plants cannot be directly applied to woody plants. In particular, as an endangered and high-value timber species, *P. bournei* has faced increasing environmental pressures in recent years, which have seriously threatened its natural distribution and long-term management. Although genome-wide analysis of the *SWEET* gene family has been conducted in other plants, the evolutionary characteristics of the *SWEET* gene family in *P. bournei*, as well as the expression mode and biological function in the hormone regulatory network, are still unknown. To this end, we identified the *SWEET* gene family in *P. bournei* using the whole-genome approach and conducted a systematic analysis of its evolutionary characteristics, hormone-responsive expression patterns, and related plant physiological responses under ABA and MeJA treatments. These analyses further reveal the functions of these genes and lay a solid foundation for more complex applications such as forest tree breeding in practice.

### 3.1. Evolutionary Conservation and Lineage-Specific Expansion of the PbSWEET Gene Family

This study identified 21 *PbSWEET* genes in the genome of *P. bournei*, which were classified into 4 subfamilies (Figure 2). This quantity is comparable to that of *O. sativa* (21) [47] and is slightly higher than that of *A. thaliana* (17) [48]. In studies on woody plants, there are 36 *Hevea brasiliensis* (Willd. ex A. Juss.) Müll. Arg. and an astonishing 52 *Eucalyptus grandis* W. Hill ex Maiden [49,50]. Generally, woody plants tend to possess more *SWEET* genes than *A. thaliana*, which may be related to the increased demand for phloem loading during wood development [51]. However, compared with other woody species, the *SWEET* gene family in *P. bournei* follows a relatively conservative expansion pattern. From the evolutionary tree, it can be seen that the *SWEET* gene family of *P. bournei* shows a non-equilibrium expansion characteristic during the evolutionary process, especially in subfamily A, where a significant expansion has occurred. Similar to this pattern, the *SWEET* gene family in *E. grandis* has also undergone a significant expansion in the B1 subgroup; this differential expansion of *E. grandis* is consistent with the need of woody plants for more sugar to be transported from the phloem to support tree growth [50], but the lineage-specific expansion mechanism and reasons in *P. bournei* are not yet clear. Notably, the expansion of the A subfamily of *P. bournei* may be closely related to its unique growth characteristics and stress adaptation strategies [52].

Chromosome localization analysis revealed that the 21 *PbSWEET* genes were unevenly distributed across 10 chromosomes (Figure 1). Among them, Chr08 contains five genes, showing a distinct clustered distribution pattern, which is highly similar to tandem repeat clusters observed in *E. grandis*, *Populus trichocarpa* Torr. & A. Gray ex Hook., *H. brasiliensis*, and *Glycine max* (L.) Merr. (both woody plants and herbaceous plants) [49,50,51,53]. It indicates that tandem duplication is an important and conserved mechanism for the expansion of the *SWEET* family. The further identified four pairs of segment duplication events (occurring between Chr01 and Chr03, Chr05 and Chr10, Chr05 and Chr12, and within Chr06) further confirmed that segment duplication was also an important driving force for the expansion of this family (Figure 5). This pattern has also been clearly mentioned in *P. trichocarpa* with five pairs of repetitive segments [51] and *G. max* with 95 genes derived from repetitive segments (both woody plants and herbaceous plants) [53]. All the detected segmentally duplicated *PbSWEET* genes had Ka/Ks < 1 (Table 2), indicating that they had undergone purifying selection, and the core sugar transport function was strictly protected during evolution. This observation is consistent with previous reports demonstrating that purifying selection is the dominant evolutionary force shaping the *SWEET* family across diverse plant lineages [49,51,53,54]. It is worth noting that the Ka/Ks value between *PbSWEET1* and *PbSWEET2* is NaN, indicating that they are the result of a recent duplication event and have not yet accumulated significant sequence differences. Similarly, in *H. brasiliensis*, *HbSWEET2e/HbSWEET2f* (Ks = 0.0085) and *HbSWEET2c/HbSWEET2d* (Ks = 0.1065) also exhibit very low Ks values for recent replicating sequences [49]. These recent replication events may provide raw materials for new functionalization or sub-functionalization and are closely related to the adaptation of species to specific habitats [55,56]. The interspecific collinearity analysis revealed that seven *PbSWEET* genes have collinearity relationships with *O. sativa*, while only four have collinearity relationships with *A. thaliana* (Figure 6). This suggests that the *SWEET* family in *P. bournei* may have retained more ancient collinearity modules with monocotyledonous plants like *O. sativa* than with *A. thaliana*. This result contrasts with the conclusion drawn in previous studies that the correlation between *Mangifera indica* L. and *A. thaliana* was more significant [57]. It may be related to the specific genomic evolutionary history experienced by different dicotyledonous woody plants. *P. bournei*’s phylogenetic position as a *Laurales*, an early-diverging angiosperm lineage, is coupled with a slow rate of genomic structural evolution and the absence of additional lineage-specific whole-genome duplications, which together preserved ancestral syntenic blocks largely lost in most eudicots.

Most of the 21 PbSWEET proteins contain the typical MtN3_slv domain (Figure 3C), and the composition of conserved motifs within the same subfamily is highly similar (Figure 3B), which is consistent with the findings in both woody plants and herbaceous plants [23,50,51,53,57]. This further confirms the high structural conservation of the SWEET family during evolution, and its core sugar transport function has a definite molecular basis [8,58]. It is worth noting that nearly half of the members in the A subfamily of *P. bournei* lack the UTR region (Figure 3D), suggesting that part of them may perform constitutive expression functions as their primary role, which is in agreement with the observations regarding the expression analysis made in the study of *Populus alba* L. × *Populus glandulosa* Uyeki (clone 84K; hereafter *Pag*) [51].

Based on the above analysis, the *SWEET* gene family exhibits characteristics of both conservation and plasticity during evolution. Its core sugar transport function is strictly protected by purification selection (manifested as the conserved MtN3 domain), but the subfamily expansion pattern and replication type (dominated by tandem repeats and segmental duplications) exhibit significant lineage specificity. This evolutionary pattern enables the *SWEET* family to not only maintain the basic sugar transport function but also to adapt to the specific physiological requirements and environmental challenges of different species.

### 3.2. Functional Prediction of PbSWEET Proteins

Subcellular localization prediction (Table 1) indicates that the majority of PbSWEET proteins are located in the plasma membrane (16), and this finding aligns with the observations in *E. grandis* (nearly 40), collectively supporting the core function of transmembrane sugar transport of this family [50,59]. GO enrichment analysis further confirmed that the PbSWEET family was significantly enriched in functions such as sugar transmembrane transport activity and carbohydrate transport (Figure 8). In addition to the predominant plasma membrane localization, some PbSWEETs are targeted to chloroplasts or vacuoles, which has also been mentioned in woody plants; for instance, PtoSWEET17b in *Populus tomentosa* Carrière is a vacuolar membrane sucrose transporter [13,46,60], and EgSWEET16a/16b in *E. grandis* are also located on the vacuolar membrane [50]. Overall, the diverse subcellular localization of the PbSWEET protein is consistent with its multiple functions in sugar transport and homeostasis regulation. Most of the members are located on the cell membrane to facilitate the transport of sugar between cells, while some subgroups targeting chloroplasts and vacuoles may regulate the intracellular sugar dynamic balance. This distribution pattern is highly consistent with the functional specialization characteristics of SWEET proteins in other woody plants, highlighting the conservation of the sugar allocation mechanism in perennial plants.

Consistent with their predicted roles in transmembrane sugar transport, the tissue-specific expression profiles of *PbSWEET* genes revealed distinct organ-specific preferences that align with known physiological demands (Figure 7). The highly expressed *PbSWEET5* in the xylem, *PbSWEET12/21* enriched in root/stem parenchyma, and *PbSWEET10/19* specifically expressed in leaves, respectively, suggest that they play a transmembrane transport role during long-distance transport in xylem tissues and the formation of secondary walls [61], sugar unloading in storage organs, and the export of photosynthetic products from source tissues. To further explore the regulatory mechanisms underlying the sugar transport function of PbSWEET proteins, PPI network prediction was performed. It indicates that PbSWEETs may interact with glycosyltransferases, sucrose transporters SUC2/SUC4, cytoskeletal component ARPC2B, PPR protein PCMP-H43, etc. (Figure 9). This suggests that it not only participates in sugar transport but may also act as an intermediary node connecting sucrose transporters to cytoskeletal transport (via ARPC2B) and glycosylation pathways; it also potentially couples sugar transport with intracellular energy metabolism via PCMP-H43. Collectively, PbSWEETs, through dynamic PPIs, act as a central hub that integrates sugar transport with intracellular transport, energy metabolism, and a broader range of physiological processes.

Taken together, the PbSWEET protein has a conserved and multifunctional role. Among them, the members located on the cell membrane are mainly responsible for the exchange of sugars between cells, while the subtypes containing cell-targeting domains can precisely regulate the sugar homeostasis within the cells. Furthermore, it is known that several interacting partners (such as SUC2/SUC4 and ARPC2B) are involved in hormone-mediated regulatory pathways, including ABA and auxin signal transduction [62,63,64], which indicates that PbSWEETs may also be regulated or participate in hormone-dependent processes. To further explore this possibility and these mechanisms, we predicted the *cis*-acting elements of the promoter regions of the *PbSWEET* genes and analyzed their expression patterns under different hormone treatment conditions.

### 3.3. Temporal Coordination of PbSWEET Expression and Plant Physiological Adjustments Under ABA and MeJA Treatments

To investigate the functional correlation of the *PbSWEET* gene under hormonal stress, we analyzed the temporal expression profiles of four candidate genes by qRT-PCR and simultaneously measured the key physiological parameters (leaf water loss rate, Pro content, soluble sugar content, soluble protein content, and MDA content) in the leaves of *P. bournei* treated with ABA or MeJA. The results show that although all four genes contain hormone-responsive *cis*-regulatory elements (Figure 4), they all exhibit significant member specificity, hormone specificity, and time-dependent expression patterns (Figure 10). This transcriptional-level difference suggests that different members of *PbSWEET* have undergone functional specialization in response to abiotic stress. It is worth noting that the dynamic changes in physiological regulation (particularly the accumulation of soluble sugars and the inhibition by Pro) (Figure 11) show a temporal coordination with the expression of specific *PbSWEET* genes, suggesting a possible functional association between the transcriptional activation of sugar transporters and the realization of the osmotic regulation mechanism.

PbSWEET10, a plasma membrane-localized bidirectional sugar transporter, exhibits high transcript abundance in leaf tissues. Under ABA treatment, the expression level gradually increased at the beginning, and then experienced a delayed explosive increase at 12 h, which coincided with the peak of soluble sugar content and the sharp increase in MDA. The gradual accumulation of soluble sugars alongside declining Pro levels is consistent with the hypothesis that early osmotic adjustment might rely primarily on sugar import. The delayed burst of *PbSWEET10* coincided with the timing of soluble sugar accumulation, and it may be speculated that this sugar influx into mesophyll cells could lower water potential and help maintain turgor under stress. However, excessive sugar influx, if it occurs, might be associated with enhanced metabolic activity, potentially contributing to reactive oxygen species (ROS) accumulation and subsequent membrane lipid peroxidation, as suggested by the MDA peak. Subsequently, *PbSWEET10* expression declines sharply, accompanied by a concurrent decrease in soluble sugar content, which is coordinated downregulation that coincides with alleviation of oxidative damage. A working hypothesis arising from this correlation is that rapid suppression of the gene could prevent excessive carbon consumption and restore cellular redox homeostasis. However, this remains a correlative inference that requires direct functional validation. The consistently higher soluble sugar levels relative to Pro further support the hypothesis that sugars function as the primary osmotic regulators under ABA-induced stress. Under the treatment of MeJA, *PbSWEET10* exhibited an explosive increase immediately at 4 h, and although it rapidly declined from the peak level afterwards, it still maintained a relatively high expression level. The soluble sugar levels rose sharply at 24 h, suggesting a possible hypothesis that the early activation of *PbSWEET10* might temporarily pump the sugar out of the cell into the periplasm to initiate defense signals, while the 24 h sugar surge might result from other transporters or metabolic changes. The bidirectional transport ability of PbSWEET10 is speculated to enable it to switch between different functions, such as osmotic regulation and defense initiation in response to stress conditions.

Although *PbSWEET10* exhibited the most intense hormone-induced expression, the second-largest member in terms of transcriptional amplitude was *PbSWEET4*, a short peptide enriched in the xylem and located in the chloroplast, lacking the classical sugar transport function. Under ABA and MeJA treatments, *PbSWEET4* exhibited an immediate and explosive transcriptional burst, followed by a sharp decline, preceding in time the subsequent oxidative burst and the late increase in soluble sugars observed under MeJA treatment. This temporal sequence raises the possibility that PbSWEET4 does not function as a sugar transporter but rather as a stress-induced signal peptide. A plausible hypothesis is that PbSWEET4 acts as a retrograde signal from the chloroplast to the nucleus in the early stage, specifically speaking, after sensing ABA or MeJA, its rapid upregulation in the parenchyma cells of the xylem could trigger changes in the redox state of the chloroplasts or the release of secondary messengers, potentially contributing to downstream defense and antioxidant responses, including the observed delayed accumulation of MDA and (under MeJA conditions) the accumulation of soluble sugars. The rapid decrease in *PbSWEET4* after its peak level might help limit the duration of signal amplification, potentially contributing to a brief and controlled stress response. Therefore, PbSWEET4 emerges as a possible atypical member of the SWEET family. It could evolve from the ancestral sugar transporter and become a signal peptide localized in the chloroplast, coordinating the early perception of stress with subsequent physiological adaptation.

PbSWEET3 is a protein that is enriched in the xylem and localized in the chloroplast, and it lacks sugar transport activity. Unlike *PbSWEET4*, which undergoes a transcriptional burst, *PbSWEET3* shows only a slight and brief increase under ABA before returning to low expression and is rapidly suppressed under MeJA. This pattern raises the possibility that *PbSWEET3* is not a signal amplifier, but rather a negative regulator of chloroplast homeostasis. A testable hypothesis is that, under normal conditions, the basic expression of *PbSWEET3* maintains the quiescence of the chloroplasts; after ABA perception, its slight increase provides a brief signal window, but quickly returns to low expression to avoid oxidative stress; under MeJA, the continuous downregulation of *PbSWEET3* can relieve the inhibition of the chloroplast defense pathway, potentially allowing for the outbreak of late MDA and a significant increase in soluble sugars. PbSWEET18 is a sugar transporter located on the plasma membrane and is expressed at low levels in all tissues. It showed a persistent and slightly upward trend under ABA, but was rapidly suppressed under MeJA (with only a brief small peak observed at 12 h). One possible interpretation is that it functions as a homeostatic transport protein, maintaining the basal glucose flux. Under ABA stress, a mild increase in activity might facilitate sugar influx, supporting the hypothesis of soluble sugar accumulation for osmotic regulation without triggering oxidative bursts. Under MeJA, it immediately reduced, potentially coinciding with restricted sugar excretion and redirected carbon allocation; the 12 h peak could be tentatively associated with short-term sugar supply for defense before the 24 h oxidative burst. In summary, PbSWEET18 could be a constitutively low-expressing transporter protein that is regulated bidirectionally through ABA induction and MeJA inhibition.

It must be emphasized that all these inferences are based on temporal concordance between gene expression and physiological changes, and should therefore be considered testable hypotheses rather than definitive conclusions. Direct functional validation—for example, through gain-of-function or loss-of-function experiments—is essential to establish causality.

**Figure 12:**
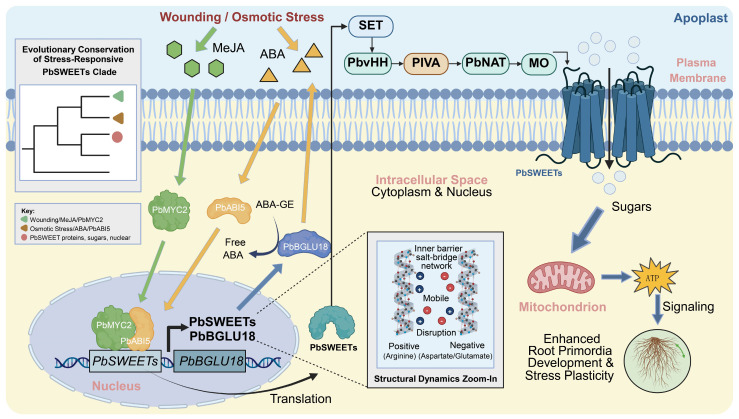
A working model of stress-responsive *PbSWEET* genes in *P. bournei*. The evolutionary tree (in the upper left corner) highlights a conserved *PbSWEET* sub-branch related to stress responses and root development. The symbols with color coding distinguish different genes associated with the wound/MeJA (green) and osmotic stress/ABA (orange) pathways. Stress signals activate the nuclear transcription factors PbMYC2 and PbABI5, which upregulate the expression of *PbSWEET* genes and *PbBGLU18*. PbBGLU18 releases free active ABA from the stored ABA-glucose esters (ABA-GE), which is then exported to the extracellular space to transmit systemic stress signals; simultaneously, the PbSWEET protein mediates transmembrane sugar transport, providing substrates for mitochondrial ATP generation, promoting root primordium development and enhancing stress plasticity. The structural magnification diagram shows how the salt bridge network stabilizes the conformational changes during sugar transmembrane transport. Arrows represent positive and negative regulatory directions.

The ANOVA analysis indicated that the induction duration of the two genes under MeJA treatment was longer, which is consistent with the persistent role of JA signaling in leaf senescence and stress adaptation. The transcriptional reprogramming activated by MeJA through the COI1-JAZ-MYC2 pathway usually involves extensive changes in metabolic flux, and sugar excretion—the core link in the redistribution of the carbon skeleton—requires the continuous expression of *SWEET* genes [65]. In contrast, the *SWEET* expression induced by ABA (especially *PbSWEET10*) has a much higher peak value, which is in line with the characteristic of ABA as a stress switch that triggers acute osmotic adjustment and transient oxidative bursts. To illustrate this regulatory divergence, we propose a working model [66,67] in which the duration and amplitude of hormonal signals are decoded into distinct transcriptional outputs of the *PbSWEETs* (Figure 12).

*P. bournei* is hypothesized to employ a carbon-centric osmotic strategy, where proline is actively inhibited. PbSWEET4 could trigger an early response as a non-translocated small peptide signal, while PbSWEET10 could perform sugar redistribution in the later stage, potentially forming a dual-wave transcriptional program. Meanwhile, a transcription–metabolism uncoupling is suggested under MeJA conditions (*PbSWEET10*), and the oxidative damage is transient, which is consistent with the interpretation that sugar transport might represent an adaptive regulation rather than passive damage. *PbSWEET10* also exhibited a specific burst-like induction under ABA treatment, which mimics certain aspects of osmotic stress signaling. Based on these observations, *PbSWEET10* emerges as a promising candidate for future functional characterization. Furthermore, we speculate that, if confirmed under actual other stress conditions, the sugar transport mediated by *PbSWEET10* might contribute to the stress resistance. This contribution could occur in the later stage of the stress via two hypothesized pathways: isolating sugar into vacuoles or cell walls to reduce water potential and minimize water loss; exporting sugar outside the mesophyll cells to transmit the acquired adaptive signals of the system. Subsequent studies will further verify the biochemical function and stress resistance contribution of *PbSWEET10* through transgenic heterologous expression, subcellular localization, and sugaromics analysis under other typical stress conditions.

## 4. Materials and Methods

### 4.1. Materials

#### 4.1.1. Genomic Data

The genomic sequence data and annotation information of *P. bournei* were obtained from the China National GeneBank (CNGB) Sequence Archive (CNSA) database (https://db.cngb.org/cnsa/, accessed on 2 January 2026), and the corresponding accession number is CNP0002030 [27]. The genome sequence files of *A. thaliana* and *O. sativa* were obtained from EnsemblPlants (https://plants.ensembl.org/, accessed on 2 January 2026) and Phytozome v13 (https://phytozome-next.jgi.doe.gov/, accessed on 2 January 2026), respectively. In addition, the RNA-seq data of different tissues of *P. bournei* were obtained from BioProject (https://www.ncbi.nlm.nih.gov/bioproject/, accessed on 2 January 2026), and the corresponding accession number is PRJNA628065.

#### 4.1.2. Plant Materials and Treatments

These plant materials were obtained from the Fujian Academy of Forestry Sciences (Fuzhou, China). One-year-old *P. bournei* seedlings were grown in an artificial climate chamber at 25 °C, 75% humidity, and a 16 h light/8 h dark photoperiod. Among these plants, we selected 27 young seedlings of *P. bournei* with basically the same growth potential and treated their leaves separately with ABA (2 mmol/L) and MeJA (2 mmol/L) for 0, 4, 8, 12, and 24 h. At the same time, photos were taken at the aforementioned time points to record the changes in the leaves of *P. bournei* over time under the treatment of ABA and MeJA in order to be used for phenotypic observation and analysis. Untreated leaves (0 h) from three biological replicates served as the control (CK). Each of the other treatments included three biological replicates. After processing, the leaf samples were collected. All the samples were immediately frozen in liquid nitrogen and then stored at −80 °C for subsequent qRT-PCR analysis and plant physiological index measurements.

### 4.2. Identification and Physicochemical Property Analysis

The conserved domains of the SWEET family in *A. thaliana* were downloaded from the Pfam database (https://www.ebi.ac.uk/interpro/entry/pfam/PF03083/, accessed on 3 January 2026). Through local BLASTp search, the conserved domains of SWEETs in *P. bournei* and *A. thaliana* were compared, and candidate SWEETs in *P. bournei* were screened out [68]. After removing the redundant results from the BLASTp search, the identified protein sequences of the *P. bournei* SWEETs were submitted to NCBI for further BLASTp search. To further identify the members of the *SWEET* gene family, the HMM model (PF03083) for the conserved domain of SWEET was downloaded from the Pfam database using the HMMER-3.2.1 software; the e-value was set to <10^−5,^ and the default parameters were used. Subsequently, the physicochemical properties of 21 PbSWEET protein sequences were analyzed using the ExPASy ProtParam online tool (https://web.expasy.org/protparam/, accessed on 4 January 2026), including predictions of parameters such as the number of amino acids, molecular weight, theoretical pI, instability index, lipid solubility index, and the GRAVY [69]. The protein subcellular localization was predicted using the WoLF PSORT online tool (https://wolfpsort.hgc.jp/, accessed on 4 January 2026) [70].

### 4.3. Chromosome Localization and Evolutionary Analysis

Using the TBtools (v2.468) software, based on the GFF of *P. bournei*, the 21 *PbSWEET* genes were located on the chromosomes, and a chromosome distribution map was drawn [71]. Using the Muscle program of MEGA11 software with default parameters, a multiple sequence alignment was conducted on the SWEET protein sequences of *P. bournei* (21), *A. thaliana* (17), and *O. sativa* (21) [72]. The complete list of *A. thaliana* and *O. sativa SWEET* genes, including their gene accession numbers, is provided (Table S2). An evolutionary tree was constructed using the maximum likelihood method with 1000 bootstrap repetitions, and the evolutionary tree was visualized and beautified using iTOL (https://itol.embl.de/, accessed on 5 January 2026) [73].

### 4.4. Gene Structure, Conserved Motifs, Conserved Domains, and Homology Modeling Analysis

Using the TBtools software, the information of gene structure, conserved domains, and conserved motifs was integrated to draw a three-in-one comprehensive analysis diagram. Among them, the gene structure was derived from the GFF annotation file of the *P. bournei* genome; the conserved domains were retrieved through the NCBI CDD database (https://www.ncbi.nlm.nih.gov/cdd/, accessed on 6 January 2026); the conserved motifs were predicted using MEME Suite (https://meme-suite.org/meme/, accessed on 6 January 2026), with the number of motifs set to 10 and all other parameters remaining at their default values [74].

The three-dimensional structure of the PbSWEET4 protein was modeled using the SWISS-MODEL workspace (https://swissmodel.expasy.org/, accessed on 2 June 2026). The template used was 5ctg.1.A, which is derived from the three-dimensional structure of the rice OsSWEET2b protein. The template was automatically screened as the optimal template based on sequence identity (48.33%) and modeling coverage. Modeling coverage was calculated as the ratio of the aligned region length to the total length of the template. After the model was constructed, the built-in tools of SWISS-MODEL were used for visualization and structural analysis.

### 4.5. Promoter Cis-Element Analysis

The 2000 bp promoter sequence upstream of the start codon of the *PbSWEET* gene was extracted, and the *cis*-acting regulatory elements were predicted and classified using the PlantCARE database (http://bioinformatics.psb.ugent.be/webtools/plantcare/html/, accessed on 6 January 2026) [75]. TBtools was used to conduct a visual display based on functional categories (light response, hormone response, stress response, growth and development-related, etc.).

### 4.6. Collinearity and Selection Pressure Analysis

The MCScanX module in the TBtools software was used to conduct intraspecific collinearity analysis of the *P. bournei* genome, identify segmental duplication events of the *PbSWEET* gene family, and present the results in the form of a Circos diagram [76]. The interspecies collinearity analysis was conducted using the One Step MCScanX module in TBtools. Comparative genomic collinearity analyses were performed for *P. bournei*, *A. thaliana*, and *O. sativa*. The species-level homology relationship of *SWEET* genes was presented through a dual synteny plot.

Using the Simple Ka/Ks Calculator (NG) module in the TBtools software, the Ka and Ks values were calculated for the duplicated gene pairs within the *PbSWEET* gene family. Furthermore, the selection pressure type of the gene pair was evaluated using the Ka/Ks ratio (Ka/Ks < 1 indicates purifying selection, Ka/Ks = 1 indicates neutral evolution, and Ka/Ks > 1 indicates positive selection) [77].

### 4.7. Tissue-Specific Expression Analysis

Based on the transcriptome data of different tissues of *P. bournei*, the expression profiles of the *PbSWEET* gene family were analyzed. Using the Heatmap module of the TBtools software, a clustering heatmap was plotted based on the expression levels converted from log_2_(FPKM + 1); both rows and columns were clustered using hierarchical clustering with Euclidean distance and the complete linkage method.

### 4.8. Functional Annotation and GO Enrichment Analysis

The functional annotation of the PbSWEET protein sequences was conducted using eggNOG-mapper (http://eggnog-mapper.embl.de/, accessed on 7 January 2026), and the GO annotation information was obtained [78]. The GO enrichment analysis of the annotation results was conducted using the TBtools software, and the results were classified and displayed according to the three major categories: biological process (BP), molecular function (MF), and cellular component (CC).

### 4.9. Prediction and Visualization of PPI Network

Based on the STRING database (https://string-db.org/, accessed on 8 January 2026), using the SWEET proteins of *A. thaliana* as a reference, the PPI network of PbSWEET proteins was predicted [79]. Using a medium confidence level parameter (0.4) as the minimum interaction score threshold, the interaction network was visualized and optimized using the Cytoscape (version 3.9) software.

### 4.10. qRT-PCR Analysis

Total RNA was extracted from both control and stress-treated samples using an RNA extraction Kit (Coolaber, Beijing, China). Then, cDNA was synthesized from the extracted RNA using the M-MLV 4 First-Strand cDNA Synthesis Kit (Biomed, Beijing, China). After that, the SYBR Green dye method was used for quantitative PCR detection. Each qRT-PCR reaction system was 20 μL, containing 10 μL of 2× SYBR Green Mix, 0.4 μL each of forward and reverse primers, 2 μL of cDNA template, and 7.2 μL of nuclease-free water. The reaction employs a three-step protocol: pre-denaturation at 95 °C for 30 s; 40 cycles (95 °C for 5 s, 60 °C for 30 s with fluorescence collection); and finally, melt curve analysis (95 °C for 15 s, 60 °C for 1 min, and 95 °C for 15 s). Each treatment and time point was set with 3 biological replicates, and each biological replicate was run with 3 technical replicates. The relative expression levels of *PbSWEET* genes were calculated using the 2^−ΔΔCT^ method, assuming that the amplification efficiencies of the target and reference genes were approximately equal and close to 100% [80,81]. The specific primers used for qRT-PCR were designed using TBtools and are listed (Table S3). The internal reference gene selected was *PbEF1α* (GenBank accession number KX682032). The stability of *EF1α* under various experimental conditions, including ABA and MeJA treatments as well as drought and osmotic stress, has been previously demonstrated in multiple plant species [82,83]. Therefore, *PbEF1α* was considered a reliable reference gene for qRT-PCR normalization in this study. The one-way ANOVA was conducted to determine whether there were significant differences among multiple time points, and Dunnett’s post hoc test was used for pairwise comparisons between each treatment time point and the 0 h control. This method was chosen because it can more accurately reflect statistical significance when dealing with data from multiple time points.

### 4.11. Physiological Index Determination

Physiological indices were determined from the same samples; for each treatment group and each time point, 3 biological replicates were set up, and 3 technical replicates were conducted for each biological replicate. A one-way ANOVA was used to test the overall differences among multiple time points, and Dunnett’s post hoc test was employed to conduct pairwise comparisons between each treatment time point (4, 8, 12, 24 h) and the 0 h control group. The measurements of all the following physiological indicators are all based on the aforementioned repetition and statistical methods.

#### 4.11.1. Determination of MDA Content

The measurement was carried out using the MDA content kit (Sinobestbio, Shanghai, China) as follows: Weigh approximately 0.1 g of the hormonally treated and frozen leaves of *P. bournei*, add 1 mL of the extraction solution, and homogenize in an ice bath. Then, perform 8000× *g* centrifugation at 4 °C for 10 min. Collect the supernatant and place on ice for measurement. Take 0.3 mL of reagent 1 and add it to a 1.5 mL centrifuge tube. Then, add 0.1 mL of the sample and mix well. Incubate in a 95 °C water bath for 30 min; then, cool it in an ice bath. Centrifuge at 10,000× *g*, 25 °C for 10 min. Finally, transfer 200 μL of the supernatant to a 96-well plate and measure the absorbance at 532 nm and 600 nm. Calculate the MDA content according to the manufacturer’s instructions.

#### 4.11.2. Determination of Soluble Protein Content

The protein content was determined using the BCA protein assay kit (Sinobestbio, Shanghai, China). Approximately 0.1 g of the hormonally treated and frozen leaves of *P. bournei* was weighed to which 1 mL of the extraction solution was added; the mixture was homogenized at 4 °C under an ice bath then centrifuged at 10,000 rpm for 10 min at 4 °C, and the supernatant was taken as the sample solution to be tested. The working solution was prepared by mixing reagent A and reagent B at a ratio of 50:1 immediately before use. Then, they were divided into three groups: blank tubes, standard tubes, and determination tubes. They were mixed in EP tubes with 200 μL of working solution and 4 μL of distilled water, 200 μL of working solution and 4 μL of standard sample, and 200 μL of working solution and 4 μL of sample to be tested. These were then placed in a 60 °C incubator for 30 min. The absorbance values were measured at 562 nm in a micro-96-well plate and recorded separately as the absorbance of the blank tube, standard tube, and sample tube. The soluble protein content was calculated according to the manufacturer’s instructions.

#### 4.11.3. Determination of Soluble Sugar Content

The determination was carried out using a plant-soluble sugar kit (Sinobestbio, Shanghai, China). Approximately 0.1 g of the frozen and hormone-treated leaves of *P. bournei* was weighed, 1 mL of distilled water was added, and the mixture was homogenized. The homogenate was transferred to a capped centrifuge tube and placed in a boiling water bath for 10 min (with the cap tightly closed to prevent evaporation). After cooling to room temperature, the mixture was centrifuged at 8000× *g* for 10 min at room temperature. The supernatant was transferred to a 10 mL test tube. It was then diluted with distilled water to 10 mL, shaken well, and set aside for use. Then, the samples were divided into three groups: blank tubes, test tubes, and standard tubes. They were mixed with 200 μL of concentrated sulfuric acid, 20 μL of working solution, and 80 μL of distilled water; 200 μL of concentrated sulfuric acid, 20 μL of working solution, 40 μL of distilled water, and 40 μL of sample; and 200 μL of concentrated sulfuric acid, 20 μL of working solution, 40 μL of distilled water, and 40 μL of standard solution in EP tubes, respectively. Then, they were placed in a 95 °C water bath for 10 min. After cooling to room temperature, 200 μL was transferred to each well of a 96-well plate. The absorbance values of the blank tube and the test tube were read at 620 nm. A standard curve was generated using glucose standards (0.0125–0.3 mg/mL) according to the kit manual, and the soluble sugar content was calculated based on the standard curve following the manufacturer’s instructions.

#### 4.11.4. Determination of Pro Content

The test was conducted using the Pro content detection kit (Sinobestbio, Shanghai, China) as follows: Weigh approximately 0.1 g of the frozen and hormone-treated leaves of *P. bournei*, and add 1 mL of the extraction solution for ice bath homogenization; then, place it in a boiling water bath for 10 min of shaking for extraction, centrifuge at 10,000× *g* at room temperature for 10 min, take the supernatant, cool it down, and wait for measurement. Take 0.25 mL of the supernatant, 0.25 mL of 4 °C glacial acetic acid, and 0.25 mL of reagent II, and mix them in a 2 mL capped EP tube. Place it in a boiling water bath and keep it there for 30 min. Shake it once every 10 min. After cooling, add 0.5 mL of 4 °C toluene, shake for 30 s, and then let it stand for a while; transfer 0.2 mL of the upper solution to a 96-well plate, measure the absorbance at a wavelength of 520 nm, and record the absorbance value. A standard curve was generated using proline standards (1–15 µg/mL) according to the kit manual, and the Pro content was calculated based on the standard curve following the manufacturer’s instructions.

#### 4.11.5. Determination of Leaf Water Loss Rate

Leaf water loss rate was determined as follows. Leaves from the 0 h control group were collected, surface water was carefully wiped off, and their size and leaf age characteristics were recorded. They were weighed to obtain the initial fresh weight (W_0_). At each subsequent time point (4, 8, 12, and 24 h), independent leaves of the same leaf age and similar size were collected and weighed (W_t_). The leaf water loss rate was calculated as (W_0_ − W_t_)/W_0_ × 100%, expressed as the percentage loss of fresh weight relative to the initial weight at 0 h.

## 5. Conclusions

This study systematically identified the SWEET gene family in *P. bournei* and revealed its evolutionary conservation and lineage-specific expansion patterns. The A subfamily was significantly expanded through tandem and segment duplication, while purifying selection strictly maintained the core sugar transport function. Most PbSWEET proteins retain the classic MtN3_slv domain, with only a few exceptions, demonstrating functional conservation.

By integrating transcriptome and physiological data analysis, this study suggests that this woody plant might possess a carbon-centered osmotic regulation strategy: Under ABA and MeJA stress, soluble sugars may replace Pro as the main osmotic regulatory substance. The time-dependent expression profile further points to a possible dual-wave transcriptional program: PbSWEET4 may act as an early stress-induced signal peptide (non-classical function), while PbSWEET10 might function as a late sugar redistributor. It is worth noting that *PbSWEET10* exhibits delayed burst-like expression under ABA treatment, synchronizing with sugar accumulation and the transient oxidation peak, making it an important candidate gene for ABA-mediated stress adaptation. Under MeJA, the decoupling phenomenon between the transcription of *PbSWEET10* and sugar accumulation suggests complex regulation at the post-transcriptional or metabolic level.

In conclusion, this study provides a comprehensive evolutionary framework for the *PbSWEET* family and nominates *PbSWEET10* as a potential key target for future functional research and molecular breeding, with the ultimate goal of enhancing the stress resistance of this endangered timber tree species. Direct functional validation is required to confirm these inferred roles.

## Figures and Tables

**Table 1:** Detailed information on 21 *PbSWEET* genes in *P. bournei* and their encoded proteins.

Gene Accession	Gene ID	Size/aa ^1^	MW ^2^/Da	Theoretical pI	Instability Index	Aliphatic Index	GRAVY	Subcellular Localization
OF15105-RA	*PbSWEET1*	239	26,550.74	8.77	31.26	118.2	0.819	Plasma membrane
OF18582-RA	*PbSWEET2*	239	26,550.74	8.77	31.26	118.2	0.819	Plasma membrane
OF29277-RA	*PbSWEET3*	145	15,589.40	9.60	43.26	102.28	0.21	Chloroplast
OF29278-RA	*PbSWEET4*	68	7869.20	8.03	39.55	90.29	0.419	Chloroplast
OF29276-RA	*PbSWEET5*	243	26,574.74	9.02	43.1	110.78	0.675	Plasma membrane
OF00547-RA	*PbSWEET6*	236	26,111.14	9.54	38.35	121.44	0.894	Vacuole
OF16634-RA	*PbSWEET7*	238	26,330.59	9.64	43.31	127.69	0.987	Plasma membrane
OF02601-RA	*PbSWEET8*	172	19,574.05	8.91	39.37	113.37	0.691	Plasma membrane
OF07621-RA	*PbSWEET9*	196	21,808.12	8.82	39.27	120.31	0.837	Vacuole
OF07626-RA	*PbSWEET10*	244	27,405.63	9.32	38.09	112.62	0.566	Plasma membrane
OF18660-RA	*PbSWEET11*	439	50,020.08	8.76	45.61	90.84	−0.178	Plasma membrane
OF24651-RA	*PbSWEET12*	259	28,611.42	9.34	30.24	127.07	0.816	Plasma membrane
OF20979-RA	*PbSWEET13*	241	26,761.05	8.77	43.12	116.85	0.85	Chloroplast
OF04050-RA	*PbSWEET14*	274	30,573.99	7.75	34.43	107.81	0.501	Plasma membrane
OF05885-RA	*PbSWEET15*	274	30,561.08	7.70	30.05	110.66	0.583	Plasma membrane
OF11473-RA	*PbSWEET16*	278	31,404.66	9.20	46.45	119.24	0.727	Plasma membrane
OF24996-RA	*PbSWEET17*	294	33,112.08	8.48	41.87	116.67	0.6	Plasma membrane
OF05961-RA	*PbSWEET18*	249	27,458.71	9.18	39.37	119.72	0.7	Plasma membrane
OF05963-RA	*PbSWEET19*	255	27,612.82	7.58	40.63	126	0.844	Plasma membrane
OF05962-RA	*PbSWEET20*	242	26,327.45	9.20	31.48	122.44	0.766	Plasma membrane
OF05964-RA	*PbSWEET21*	297	32,377.34	9.64	36.58	114.88	0.354	Plasma membrane

^1^ aa: amino acid number; ^2^ MW: molecular weight.

**Table 2:** Pairwise evolutionary selection pressure analysis of *PbSWEET* genes.

Sequence 1	Sequence 2	Ka ^1^	Ks ^2^	Ka/Ks ^3^	Effective Length	S-Sites ^4^	N-Sites ^5^	cN ^6^	cS ^7^	pN ^8^	pS ^9^
*PbSWEET1*	*PbSWEET2*	0	0	NaN ^10^	717	175	542	0	0	0	0
*PbSWEET6*	*PbSWEET8*	0.27	1.03	0.261	516	124	392	88.75	69.25	0.226	0.560
*PbSWEET6*	*PbSWEET7*	0.11	0.43	0.259	705	175	530	54.75	57.25	0.103	0.327
*PbSWEET17*	*PbSWEET16*	0.25	0.82	0.307	828	196	632	135.17	97.83	0.214	0.499

^1^ Ka: nonsynonymous substitution rate; ^2^ Ks: synonymous substitution rate; ^3^ Ka/Ks: nonsynonymous-to-synonymous substitution ratio; ^4^ S-sites: average number of synonymous sites; ^5^ N-sites: average number of nonsynonymous sites; ^6^ cN: corrected number of nonsynonymous substitutions; ^7^ cS: corrected number of synonymous substitutions; ^8^ pN: proportion of nonsynonymous differences; ^9^ pS: proportion of synonymous differences; ^10^ NaN: not a number.

## Data Availability

The original contributions presented in the study are included in the article. The genomic and transcriptomic datasets analyzed in this study are available in public repositories (CNGB: CNP0002030; BioProject: PRJNA628065). Further inquiries can be directed to the corresponding authors.

## Supplementary Materials

The following supporting information can be downloaded at: https://www.mdpi.com/article/10.3390/plants15121914/s1, Figure S1: Homology modeling of PbSWEET4; Table S1: Summary of domain coordinates and E-values for each PbSWEET protein; Table S2: Accession numbers of *SWEET* genes from *Arabidopsis thaliana* and *Oryza sativa* used in evolutionary analysis; Table S3: Primer sequences used for qRT-PCR analysis.

## Abbreviations

The following abbreviations are used in this manuscript:

ABA	Abscisic acid
ABA-GE	ABA-glucose esters
ABRE	ABA-responsive element
ARE	Anaerobic induction element
BP	Biological Process
CC	Cellular Component
CK	Control
G-Box	CACGTG core motif
GA	Gibberellic acid
GO	Gene Ontology
GRAVY	Grand average of hydropathicity
GUS	β-Glucuronidase
JA	Jasmonic acid
Ka	Nonsynonymous substitution rate
Ka/Ks	Ratio of nonsynonymous to synonymous substitution rate
Ks	Synonymous substitution rate
MDA	Malondialdehyde
MeJA	Methyl jasmonate
MF	Molecular Function
MtN3	*Medicago truncatula* Nodulin 3
MtN3_slv	*Medicago truncatula* Nodulin 3/saliva domain
PPI	Protein–protein interaction
PPR	Pentatricopeptide repeat (protein)
Pro	Proline
ROS	Reactive oxygen species
SP	Soluble protein
SS	Soluble sugar
SUC	Sucrose transporter
SWEET	Sugars Will Eventually be Exported Transporters

## References

[1] Keller I., Neuhaus H. (2025). Humboldt review: Function and characterization of sugar transport across plant membranes: History and perspectives. J. Plant Physiol..

[2] Guo W., Pommerrenig B., Neuhaus H., Keller I. (2023). Interaction between sugar transport and plant development. J. Plant Physiol..

[3] Andersen C., Bavnhoj L., Brag S., Bohush A., Chrenková A., Driller J., Pedersen B. (2025). Comparative analysis of STP6 and STP10 unravels molecular selectivity in sugar transport proteins. Proc. Natl. Acad. Sci. USA.

[4] Li X., Ren Z., Zhang Z., Liu Y., He K., Zhang F., Guo J., Wei S., Yang D., Li W. (2025). Evolutionary trajectory and functional diversity of SWEET sugar transporters in plants. Plant J..

[5] Bhagat S., Sahoo A., Meena M., Swapnil P. (2026). Molecular mechanisms and evolutionary adaptations of transporters for photosynthates and specialized metabolites in plants. Plant Cell Rep..

[6] Liu H., Gao X., Fan W., Fu X. (2025). Optimizing carbon and nitrogen metabolism in plants: From fundamental principles to practical applications. J. Integr. Plant Biol..

[7] Breia R., Conde A., Badim H., Fortes A., Gerós H., Granell A. (2021). Plant SWEETs: From sugar transport to plant-pathogen interaction and more unexpected physiological roles. Plant Physiol..

[8] Ji J., Yang L., Fang Z., Zhang Y., Zhuang M., Lv H., Wang Y. (2022). Plant SWEET Family of Sugar Transporters: Structure, Evolution and Biological Functions. Biomolecules.

[9] Zhao J., Wang J., Liu J., Zhang P., Kudoyarova G., Liu C., Zhang K. (2024). Spatially distributed cytokinins: Metabolism signaling, and transport. Plant Commun..

[10] Chen Y., Miller A., Qiu B., Huang Y., Zhang K., Fan G., Liu X. (2024). The role of sugar transporters in the battle for carbon between plants and pathogens. Plant Biotechnol. J..

[11] Singh J., Das S., Gupta K., Ranjan A., Foyer C., Thakur J. (2023). Physiological implications of SWEETs in plants and their potential applications in improving source-sink relationships for enhanced yield. Plant Biotechnol. J..

[12] Chen J., Sun M., Xiao G., Shi R., Zhao C., Zhang Q., Yang S., Xuan Y. (2023). Starving the enemy: How plant and microbe compete for sugar on the border. Front. Plant Sci..

[13] Yang Q., Wang G., Dou J., An W., Zhang Y., Wang S., Tang Z., Yu J. (2025). Plant Sugar Transporters SWEETs: Structure, Function, and Cutting-Edge Research Insights. J. Agric. Food Chem..

[14] Ren Y., Liao S., Xu Y. (2023). An update on sugar allocation and accumulation in fruits. Plant Physiol..

[15] Zhu C., Jing B., Lin T., Li X., Zhang M., Zhou Y., Yu J., Hu Z. (2024). Phosphorylation of sugar transporter TST2 by protein kinase CPK27 enhances drought tolerance in tomato. Plant Physiol..

[16] Zhu Y., Tian Y., Han S., Wang J., Liu Y., Yin J. (2024). Structure, evolution, and roles of SWEET proteins in growth and stress responses in plants. Int. J. Biol. Macromol..

[17] Loo E., Durän P., Pang T., Westhoff P., Deng C., Durän C., Lercher M., Garrido-Oter R., Frommer W. (2024). Sugar transporters spatially organize microbiota colonization along the longitudinal root axis of Arabidopsis. Cell Host Microbe.

[18] Yang J., Luo D., Yang B., Frommer W., Eom J. (2018). SWEET11 and 15 as key players in seed filling in rice. New Phytol..

[19] Kanno Y., Oikawa T., Chiba Y., Ishimaru Y., Shimizu T., Sano N., Koshiba T., Kamiya Y., Ueda M., Seo M. (2016). AtSWEET13 and AtSWEET14 regulate gibberellin-mediated physiological processes. Nat. Commun..

[20] Anfang M., Shani E. (2021). Transport mechanisms of plant hormones. Curr. Opin. Plant Biol..

[21] Zhu J., Zhou L., Li T., Ruan Y., Zhang A., Dong X., Zhu Y., Li C., Fan J. (2022). Genome-Wide Investigation and Characterization of SWEET Gene Family with Focus on Their Evolution and Expression during Hormone and Abiotic Stress Response in Maize. Genes.

[22] Zeng Z., Lyu T., Lyu Y. (2022). *LoSWEET14*, a Sugar Transporter in Lily, Is Regulated by Transcription Factor *LoABF2* to Participate in the ABA Signaling Pathway and Enhance Tolerance to Multiple Abiotic Stresses in Tobacco. Int. J. Mol. Sci..

[23] Song W., Xue L., Jin X., Liu X., Chen X., Wu X., Cui M., Liu Q., Wang D. (2025). Genome-wide identification of *SWEET* family genes and functional analysis of *NtSWEET12i* under drought and saline-alkali stresses in tobacco. BMC Plant Biol..

[24] Ren R., Chen Y., Yu X., Peng X., Zeng L., Fang T. (2025). Identification and characterization of SWEET gene family in passion fruit reveals the involvement of *PeSWEET3* in soluble sugar accumulation. Plant Physiol. Biochem..

[25] Zou X., Du B., Zhou J., Hu J., Cao Y., Zhang L. (2025). Functional Studies and Expression Characteristics of the Vacuolar Sugar Transporter CoSWEET2a in *Camellia oleifera*. Plants.

[26] Yao L., Li S., Zhou N., Guo Y. (2025). The Mechanism of Seed Priming with Abscisic Acid for Enhancing Cuticle Deposition Under Drought Stress: Phenotypic and Transcriptomic Insights. Agriculture.

[27] Han X., Zhang J., Han S., Li Chong S., Meng G., Song M., Wang Y., Zhou S., Liu C., Lou L. (2022). The chromosome-scale genome of *Phoebe bournei* reveals contrasting fates of terpene synthase (TPS)-a and TPS-b subfamilies. Plant Commun..

[28] Li Y., Chen Y., Li P., Huang H., Xue K., Cai S., Liao X., Jin S., Zheng D. (2024). Microplastics in soil affect the growth and physiological characteristics of Chinese fir and Phoebe bournei seedlings. Environ. Pollut..

[29] Yu J., Yin K., Liu Y., Li Y., Zhang J., Han X., Tong Z. (2024). Co-expression network analysis reveals PbTGA4 and PbAPRR2 as core transcription factors of drought response in an important timber species *Phoebe bournei*. Front. Plant Sci..

[30] Li X., Liu L., Sun S., Li Y., Jia L., Ye S., Yu Y., Dossa K., Luan Y. (2022). Leaf-transcriptome profiles of *phoebe bournei* provide insights into temporal drought stress responses. Front. Plant Sci..

[31] Liu Y., Li X., Ni M., Zhang Y., Ma W., Song Y., Tong Z., Zhang J. (2025). PbHDZ35, an HD-ZIP transcription factor, regulates the trade-off between growth and drought stress in Phoebe bournei. Tree Physiol..

[32] Lian Y., Peng L., Shi X., Zheng Q., Fan D., Feng Z., Liu X., Ma H., Cao S., Chang W. (2025). Genome-Wide Identification of *GLK* Family Genes in *Phoebe bournei* and Their Transcriptional Analysis Under Abiotic Stresses. Int. J. Mol. Sci..

[33] Ma Y., Zhong M., Li J., Jiang Y., Zhou X., Ijeoma C., Tang X., Chen S., Cao S. (2023). Genome Identification and Evolutionary Analysis of *LBD* Genes and Response to Environmental Factors in *Phoebe bournei*. Int. J. Mol. Sci..

[34] Zheng K., Lu J., He X., Lan S., Zhai T., Cao S., Lin Y. (2024). Genome-Wide Identification and Expression Analysis of *GATA* Family Genes in *Dimocarpus longan* Lour. Int. J. Mol. Sci..

[35] Fakher B., Jakada B., Greaves J., Wang L., Niu X., Cheng Y., Zheng P., Aslam M., Qin Y., Wang X. (2022). Identification and expression analysis of pineapple sugar transporters reveal their role in the development and environmental response. Front. Plant Sci..

[36] Xie N., Li B., Yu J., Shi R., Zeng Q., Jiang Y., Zhao D. (2022). Transcriptomic and proteomic analyses uncover the drought adaption landscape of Phoebe zhennan. BMC Plant Biol..

[37] Bezrutczyk M., Yang J., Eom J., Prior M., Sosso D., Hartwig T., Szurek B., Oliva R., Vera-Cruz C., White F. (2018). Sugar flux and signaling in plant-microbe interactions. Plant J..

[38] Julius B., Leach K., Tran T., Mertz R., Braun D. (2017). Sugar Transporters in Plants: New Insights and Discoveries. Plant Cell Physiol..

[39] González-Suárez P. (2026). The future is sweet: Exploiting sugar allocation and use for cotton breeding. Plant Cell.

[40] Liu D., Zhang P., Zhou T., Wu Y., Yuan M., Zhang X., Liu Y. (2025). Genome-wide characterization and expression analysis of the *bHLH* gene family in response to abiotic stresses in *Zingiber officinale* Roscoe. BMC Genom..

[41] Jeena G., Kumar S., Shukla R. (2019). Structure, evolution and diverse physiological roles of SWEET sugar transporters in plants. Plant Mol. Biol..

[42] Gautam T., Dutta M., Jaiswal V., Zinta G., Gahlaut V., Kumar S. (2022). Emerging Roles of SWEET Sugar Transporters in Plant Development and Abiotic Stress Responses. Cells.

[43] Saudek V. (2012). Cystinosin, MPDU1, SWEETs and KDELR Belong to a Well-Defined Protein Family with Putative Function of Cargo Receptors Involved in Vesicle Trafficking. PLoS ONE.

[44] Wang Y., Tan B. (2025). Pentatricopeptide repeat proteins in plants: Cellular functions, action mechanisms, and potential applications. Plant Commun..

[45] Xue X., Li J., Yu Y., Chen L. (2026). SWEET1-mediated glucose transport is crucial for energy availability in Arabidopsis. New Phytol..

[46] Geng Y., Wu M., Zhang C. (2020). Sugar Transporter ZjSWEET2.2 Mediates Sugar Loading in Leaves of *Ziziphus jujuba* Mill. Front. Plant Sci..

[47] Yuan M., Zhao J., Huang R., Li X., Xiao J., Wang S. (2014). Rice *MtN3*/*saliva*/*SWEET* gene family: Evolution, expression profiling, and sugar transport. J. Integr. Plant Biol..

[48] Chen L., Hou B., Lalonde S., Takanaga H., Hartung M., Qu X., Guo W., Kim J., Underwood W., Chaudhuri B. (2010). Sugar transporters for intercellular exchange and nutrition of pathogens. Nature.

[49] Sui J., Xiao X., Qi J., Fang Y., Tang C. (2017). The SWEET gene family in *Hevea brasiliensis*—its evolution and expression compared with four other plant species. FEBS Open Bio.

[50] Yin Q., Zhu L., Du P., Fan C., Wang J., Zhang B., Li H. (2020). Comprehensive analysis of *SWEET* family genes in *Eucalyptus* (*Eucalyptus grandis*). Biotechnol. Biotechnol. Equip..

[51] Zhang L., Wang L., Zhang J., Song C., Li Y., Li J., Lu M. (2021). Expression and localization of SWEETs in *Populus* and the effect of *SWEET7* overexpression in secondary growth. Tree Physiol..

[52] Rani S., Zahra R., Bakar A., Rizwan M., Sultan A., Zain M., Mehmood A., Danial M., Shakoor S., Saleem F. (2023). Dynamic Evolution of *NLR* Genes in Dalbergioids. Genes.

[53] Patil G., Valliyodan B., Deshmukh R., Prince S., Nicander B., Zhao M., Sonah H., Song L., Lin L., Chaudhary J. (2015). Soybean (*Glycine max*) SWEET gene family: Insights through comparative genomics, transcriptome profiling and whole genome re-sequence analysis. BMC Genom..

[54] Han X., Han S., Zhu Y., Liu Y., Gao S., Yin J., Wang F., Yao M. (2023). Genome-Wide Identification and Expression Analysis of the SWEET Gene Family in *Capsicum annuum* L.. Int. J. Mol. Sci..

[55] Wilson J., Bieker V., van Boheemen L., Connallon T., Martin M., Battlay P., Hodgins K. (2025). Copy number variation contributes to parallel local adaptation in an invasive plant. Proc. Natl. Acad. Sci. USA.

[56] Hou J., Liu M., Yang K., Liu B., Liu H., Liu J. (2025). Genetic variation for adaptive evolution in response to changed environments in plants. J. Integr. Plant Biol..

[57] Zhou L., Liu X., Leng X., Zhang M., Yang Z., Xu W., Wang S., Wu H., Liang Q. (2025). Genome-Wide Identification and Expression Analysis of the Mango (*Mangifera indica* L.) SWEET Gene Family. Horticulturae.

[58] Ding R., Xiao T., Li S., Qiang J., Zhang H., Chang H., Yan Y., Li X. (2025). Wheat endosperm-specific transcription factor TaDOF6 enhances grain development by regulating *TaSWEET13h* expression and facilitating sugar and gibberellin transport. Front. Plant Sci..

[59] Zhang X., Feng C., Wang M., Li T., Liu X., Jiang J. (2021). Plasma membrane-localized SlSWEET7a and SlSWEET14 regulate sugar transport and storage in tomato fruits. Hortic. Res..

[60] Lu J., Wang Y., Wen Y., Ren Q., Wang Q., Wang X., Liu C., Zhang Q., Luo K. (2025). A ray localized vacuolar sucrose transport is required for wood formation in *Populus tomentosa*. Plant J..

[61] Hao X., Li J., Zhang L., Fan Z., Wang Z., Hou L., Wang L. (2024). PagSWEET17a mediates sucrose allocation to xylem during wood formation in poplar. Ind. Crops Prod..

[62] Gong X., Liu M., Zhang L., Ruan Y., Ding R., Ji Y., Zhang N., Zhang S., Farmer J., Wang C. (2015). Arabidopsis *AtSUC2* and *AtSUC4*, encoding sucrose transporters, are required for abiotic stress tolerance in an ABA-dependent pathway. Physiol. Plant..

[63] Ren Y., Zhang Z., Zhanakhmetova D., Li W., Chen S., Werner T., Liesche J. (2024). Fast and simple fluorometric measurement of phloem loading exposes auxin-dependent regulation of Arabidopsis sucrose transporter *AtSUC2*. Plant J..

[64] García-González J., Kebrlová S., Semerák M., Lacek J., Baby I., Petrásek J., Schwarzerová K. (2020). Arp2/3 Complex Is Required for Auxin-Driven Cell Expansion Through Regulation of Auxin Transporter Homeostasis. Front. Plant Sci..

[65] Zhou K., Han T., Pan B., Hu X., Chen X., Liu X., Fei S., Yang Y., Li W., Du M. (2025). Robustness in jasmonate signaling: Mechanisms of concerted regulation and implications for crop improvement. aBIOTECH.

[66] Kazan K., Manners J. (2013). MYC2: The Master in Action. Mol. Plant.

[67] Ondzighi-Assoume C., Chakraborty S., Harris J. (2016). Environmental Nitrate Stimulates Abscisic Acid Accumulation in Arabidopsis Root Tips by Releasing It from Inactive Stores. Plant Cell.

[68] Zhao X., Liu D., Wang Q., Ke S., Li Y., Zhang D., Zheng Q., Zhang C., Liu Z., Lan S. (2022). Genome-wide identification and expression analysis of the GRAS gene family in *Dendrobium chrysotoxum*. Front. Plant Sci..

[69] Gasteiger E., Gattiker A., Hoogland C., Ivanyi I., Appel R., Bairoch A. (2003). ExPASy: The proteomics server for in-depth protein knowledge and analysis. Nucleic Acids Res..

[70] Horton P., Park K., Obayashi T., Fujita N., Harada H., Adams-Collier C., Nakai K. (2007). WoLF PSORT: Protein localization predictor. Nucleic Acids Res..

[71] Chen C., Chen H., Zhang Y., Thomas H., Frank M., He Y., Xia R. (2020). TBtools: An Integrative Toolkit Developed for Interactive Analyses of Big Biological Data. Mol. Plant..

[72] Kumar S., Stecher G., Li M., Knyaz C., Tamura K. (2018). MEGA X: Molecular Evolutionary Genetics Analysis across Computing Platforms. Mol. Biol. Evol..

[73] Letunic I., Bork P. (2021). Interactive Tree Of Life (iTOL) v5: An online tool for phylogenetic tree display and annotation. Nucleic Acids Res..

[74] Bailey T., Johnson J., Grant C., Noble W. (2015). The MEME Suite. Nucleic Acids Res..

[75] Rombauts S., Déhais P., Van Montagu M., Rouzé P. (1999). PlantCARE, a plant *cis*-acting regulatory element database. Nucleic Acids Res..

[76] Wang Y., Tang H., DeBarry J., Tan X., Li J., Wang X., Lee T., Jin H., Marler B., Guo H. (2012). *MCScanX*: A toolkit for detection and evolutionary analysis of gene synteny and collinearity. Nucleic Acids Res..

[77] Yang Z. (2007). PAML 4: Phylogenetic analysis by maximum likelihood. Mol. Biol. Evol..

[78] Cantalapiedra C., Hernández-Plaza A., Letunic I., Bork P., Huerta-Cepas J. (2021). eggNOG-mapper v2: Functional Annotation, Orthology Assignments, and Domain Prediction at the Metagenomic Scale. Mol. Biol. Evol..

[79] Szklarczyk D., Gable A., Lyon D., Junge A., Wyder S., Huerta-Cepas J., Simonovic M., Doncheva N., Morris J., Bork P. (2019). STRING v11: Protein-protein association networks with increased coverage, supporting functional discovery in genome-wide experimental datasets. Nucleic Acids Res..

[80] Pfaffl M. (2001). A new mathematical model for relative quantification in real-time RT-PCR. Nucleic Acids Res..

[81] Livak K., Schmittgen T. (2001). Analysis of relative gene expression data using real-time quantitative PCR and the 2^−ΔΔ*C*^_T_ method. Methods.

[82] Lv W., Yang H., Zheng Q., Liao W., Chen L., Lian Y., Lin Q., Huo S., Rehman O., Liu W. (2024). Identification and Expression Analysis of TCP Transcription Factors Under Abiotic Stress in *Phoebe bournei*. Plants.

[83] Martins G., Freitas N., Máximo W., Paiva L. (2018). Gene expression in two contrasting hybrid clones of *Eucalyptus camaldulensis* x *Eucalyptus urophylla* grown under water deficit conditions. J. Plant Physiol..

