# Supplementary material for: Genome-Wide Identification and Characterization of the SWEET Gene Family in Phoebe bournei with an Emphasis on Hormonal Responses and Plant Physiological Changes
Source: Plants (Basel). 2026 Jun 20;15(12):1914. doi: 10.3390/plants15121914 (PMC13307037; doi:10.3390/plants15121914)
Supplement: Supplementary file 1 [file plants-15-01914-s001.zip › Supplementary_Figure S1_Homology modeling_of_PbSWEET4..pdf]

Supplementary Figure S1 Homology modeling of PbSWEET4.

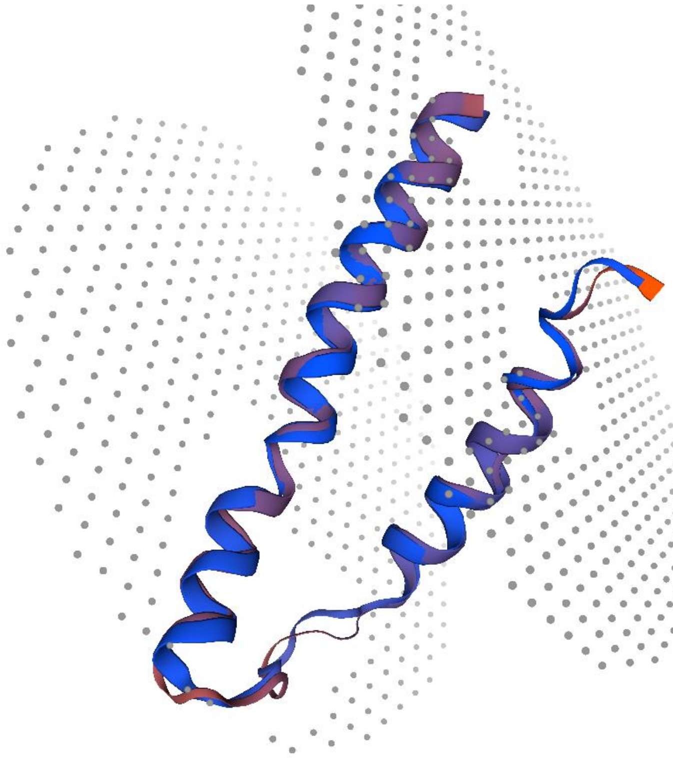

Figure S1. Predicted 3D structure of PbSWEET4. The 68-amino-acid PbSWEET4 protein was modeled using SWISS-MODEL with rice OsSWEET2b (PDB ID: 5ctg.1.A) as the template, showing a sequence identity of 48.33% and a modeling coverage of 20%–30%. This short protein contains only two helices and is classified as the MtN3_slv superfamily domain.
